# Distinct Skin Penetration and Immune Responses to Ionic and Nanoparticulate Cobalt in Allergic Contact Dermatitis

**DOI:** 10.1111/all.70422

**Published:** 2026-06-20

**Authors:** Paulina Werner, Martin Schaier, Gabriel Braun, Anneli Julander, Klara Midander, Marlene Isaksson, Gunda Koellensperger, Lukas Wisgrill, Nanna Fyhrquist

**Affiliations:** ^1^ Institute of Environmental Medicine Karolinska Institutet Solna Sweden; ^2^ Institute of Analytical Chemistry, Faculty of Chemistry University of Vienna Vienna Austria; ^3^ Vienna Doctoral School in Chemistry (DoSChem) University of Vienna Vienna Austria; ^4^ IVL Swedish Environmental Research Institute Stockholm Sweden; ^5^ Department of Occupational and Environmental Dermatology Skåne University Hospital Malmö, Lund University Malmö Sweden; ^6^ Exposome Austria, Research Infrastructure and National EIRENE Hub Vienna Austria; ^7^ Division of Neonatology, Pediatric Intensive Care and Neuropediatrics, Comprehensive Center for Pediatrics Medical University of Vienna Vienna Austria; ^8^ Department of Health Sciences Karlstad University Karlstad Sweden

**Keywords:** allergic contact dermatitis, cobalt nanoparticles, interferon signaling, metal speciation, skin penetration

## Abstract

**Background:**

Cobalt is a well‐established cause of allergic contact dermatitis (ACD), but data on the skin absorption, toxicity, and immunological effects of cobalt nanoparticles (CoNPs) remain limited. Given the increasing use of nanomaterials in industry and medicine, understanding how cobalt speciation affects skin immune activation is of growing clinical relevance.

**Methods:**

Fourteen cobalt‐allergic individuals underwent standardized patch testing with cobalt chloride (CoCl_2_) and CoNPs at equivalent nominal doses. Skin biopsies from positive reactions were analyzed by RNA sequencing to characterize immune and barrier‐related pathways. Cobalt penetration and localization were further examined in reconstructed and ex vivo human skin models using inductively coupled plasma mass spectrometry (ICP‐MS) and laser ablation ICP‐TOFMS imaging.

**Results:**

Both CoCl_2_ and CoNPs elicited positive patch test reactions, although CoNP responses were delayed and milder. CoCl_2_ also demonstrated a clearer dose‐dependent increase in clinical reactivity, whereas CoNP responses were comparatively stable across concentrations. Transcriptomic profiling revealed substantial overlap between exposures, with reaction strength emerging as the main determinant of gene expression changes. Increasing severity was associated with enrichment of interferon signaling, inflammatory response, apoptosis, cell cycle‐related programs, and glycolysis in both exposures. Lipid metabolic pathways were significantly associated with reaction strength in CoCl_2_ ‐exposed skin but not in CoNP‐exposed samples. Direct comparison using a paired DESeq2 design identified 281 differentially expressed genes between CoCl_2_ and CoNP. Functional enrichment of these genes revealed distinct biological signatures between exposures, including upregulated interferon and antiviral pathways and downregulated epithelial differentiation programs in CoCl_2_ compared to CoNP. In complementary skin models, CoCl_2_ exhibited deeper penetration, higher trans‐epidermal flux, and broader intracellular accumulation across epidermal and dermal cell populations, whereas CoNPs remained largely confined to the stratum corneum and adnexal structures.

**Conclusions:**

Cobalt‐induced ACD is shaped by both reaction strength and cobalt speciation. While CoCl_2_ and CoNPs share a core interferon‐driven inflammatory program that scales with clinical severity, ionic cobalt penetrates more deeply and preferentially engages interferon and antiviral pathways in dermal compartments, whereas CoNPs are retained more superficially and are associated with epithelial differentiation programs. These findings underscore the importance of chemical form, tissue penetration, bioavailability, and retention in determining immune responses and dermal hazard of cobalt and other metal nanomaterials.

## Graphical abstract

Cobalt chloride (CoCl_2_) and cobalt nanoparticles (CoNPs) induce a shared, severity‐dependent inflammatory response in cobalt‐sensitized skin characterized by activation of interferon signaling, inflammatory pathways (TNF/NF‐κβ, IL6‐STAT3), apoptosis/UPR, cell cycle programs, and glycolysis. Despite comparable nominal exposure, the two cobalt forms exhibit distinct tissue distribution: CoCl_2_ penetrates deeply and accumulates intracellularly across epidermal and dermal compartments, whereas CoNPs are largely retained in the stratum corneum. Direct comparison reveals divergent transcriptional programs, with CoCl_2_ preferentially inducing interferon and antiviral responses (e.g., IRF7), and CoNPs promoting keratinization and epithelial differentiation (e.g., KRT25). Together, these findings demonstrate that cobalt speciation and penetration depth shape tissue exposure and downstream immune responses in allergic contact dermatitis.

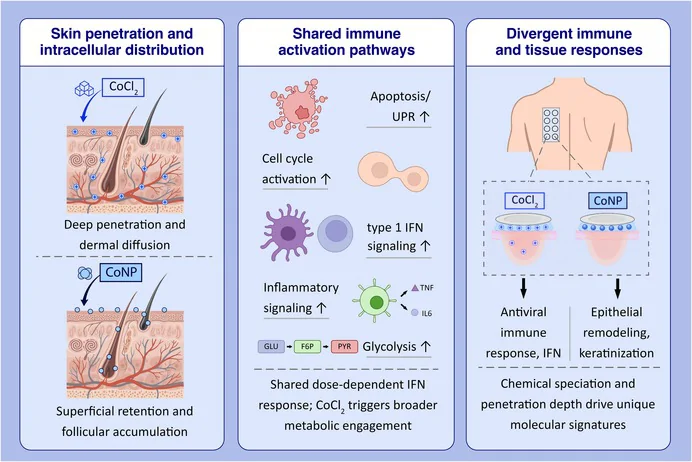

**Abbreviations**
CoCl_2_

: cobalt chloride
CoNPs
: cobalt nanoparticles
IFN
: interferon
IL
: interleukin
IRF
: interferon regulatory factor 7
KRT25
: keratin 25
NF‐κB
: nuclear factor‐κB
STAT3
: signal transducer and activator of transcription
TNF
: tumor necrosis factor
UPR
: unfolded protein response

## 1 Introduction

Cobalt is the second most common metal responsible for contact allergy in industrialized countries, contributing to 1%–4% of all cases in the general population [1]. In dermatitis patients, the prevalence is even higher, 4%–11%^2^. In occupational settings, cobalt allergy is prevalent across several sectors, including electronics manufacturing, healthcare, construction, and hard metal industries [2]. Workplace exposure to cobalt typically occurs via inhalation and direct skin contact. Notably, hard metal workers are exposed to particularly high levels, as highlighted by a study in which most participants had detectable levels of cobalt in their urine, even in the absence of any significant concentrations in air [3].

Cobalt‐based materials are extensively utilized across multiple industrial sectors, with the development of nanotechnology broadening their range of applications. Cobalt nanoparticles (CoNPs) are used in drug delivery systems, magnetic resonance imaging, and in energy storage devices [4]. In human biology, cobalt is an essential trace element, serving as a key component of vitamin B12, which is vital for numerous physiological processes [5]. However, while small amounts of cobalt are necessary for health, excessive exposure can be toxic. Well‐established toxic effects of cobalt metal and its soluble cobalt (II) salts include damage to the heart, thyroid, liver, kidneys, as well as skin sensitization, pulmonary fibrosis, and, more recently, classification as probably carcinogenic to humans (Group 2A, IARC) [6]. In contrast, the immunomodulatory effects, sensitization potential, and skin penetration properties of CoNPs remain poorly understood.

The immunological pathways driving cobalt‐induced allergic sensitization are still not fully defined. Like nickel, cobalt ions are proposed to bind directly to histidine residues on the dimerization interface of toll‐like receptor 4 (TLR4), triggering proinflammatory signaling pathways such as NF‐κB activation [7, 8]. Additionally, cobalt metal ions may induce reactive oxygen species (ROS) in human keratinocytes in vitro, potentially leading to extracellular matrix (ECM) degradation and ATP release [9], thereby contributing to a proinflammatory microenvironment in the skin. Concerns have also been raised regarding the immunological impact of nanoparticles, as their high surface area may enhance local immune activation in sensitized individuals [10]. With the global shift toward sustainable technologies, such as electric vehicles and battery production, the demand for cobalt is expected to rise significantly [11]. In light of increasing risk of exposure, detailed investigations into the specific effects of CoNP contact with human skin are timely and necessary.

Although research on the potential risk of cobalt nanomaterials is ongoing, significant knowledge gaps remain regarding their effects following skin exposure. We recently demonstrated that topical application of CoNPs via patch testing can elicit allergic contact dermatitis (ACD) in individuals with confirmed cobalt allergy [12]. In the same study, cobalt was detected in gluteal skin following CoNP application, indicating dermal penetration. Similarly, in vitro studies using Franz diffusion cells with ex vivo human skin have shown that both metallic CoNPs and cobalt oxide nanoparticles (Co_3_O_4_NP) can penetrate the skin barrier, potentially contributing to systemic exposure [13, 14]. Further supporting this, a recent study by Hagvall et al. [15], reported substantial cobalt penetration into the epidermis and deeper dermal layers following exposure to cobalt (II) metal. Despite these findings, the specific skin structures involved in cobalt accumulation and the mechanisms of interaction between cobalt and immune cells remain poorly understood.

In this study, we investigated the uptake and biological effects of CoNPs on human skin, using CoCl_2_—a well‐established reference compound for cobalt contact allergy as a comparator. We analyzed cutaneous gene expression profiles in individuals with cobalt allergy using RNA sequencing (RNA‐seq), while the uptake and spatial distribution of cobalt in healthy human skin were assessed using laser ablation inductively coupled plasma time‐of‐flight mass spectrometry (LA‐ICP‐TOFMS). The primary objectives were to characterize transcriptional changes associated with cobalt‐induced inflammation and to map the uptake patterns of cobalt in intact human skin, thereby providing mechanistic insights essential for evaluating the potential health risks of CoNP exposure.

## 2 Method

### 2.1 Recruitment of Study Participants and Sample Collection

Recruitment of study participants (*n* = 14) and patch testing were performed by a dermatologist at the Clinic of Occupational and Environmental Dermatology at Skåne University Hospital, using four concentrations of cobalt (10, 25, 50 and 100 μg/cm^2^) in the form of CoNP and CoCl_2_ applied in parallel chambers, together with vehicle controls, as previously described [12]. In brief, patch testing was conducted using CoNPs (uncoated, metal purity 99.8%, < 100 nm, CAS: 7440‐48‐4, American Elements, Los Angeles, USA) and CoCl_2_ (hexahydrate, CoCl_2_·6H_2_O, CAS: 7791‐13‐1, Merck KGaA, Darmstadt, Germany), diluted in ultrapure water to surface doses of 0, 10, 25, 50 and 100 μg/cm^2^, expressed as equivalent cobalt content irrespective of chemical form, in accordance with the European Society of Contact Dermatitis guidelines [16]. The applied doses represented defined surface loadings relevant for dermal exposure scenarios. The control condition consisted of an aluminum Finn Chamber (12 mm; SmartPractice, Phoenix USA) containing filter paper soaked with the same volume of ultrapure water as used for the CoCl_2_ and CoNP exposures, thereby controlling for both chamber conditions and vehicle exposure. The CoNPs used in this study have previously been thoroughly characterized, including size distribution by transmission electron microscopy [4, 12].

Patch tests were removed after 48 h, and reactions were evaluated by a dermatologist at 48, 96, and 168 h in accordance with European guidelines [16]. Differences in clinical reactivity to CoCl_2_ and CoNPs within the same individual were assessed by the Wilcoxon matched‐pair signed‐rank test.

Skin punch biopsies (3 mm) were obtained from patch test sites showing positive reactions or, in the absence of a reaction, from the site exposed to the highest dose, as well as from the control site. Three biopsies (control, CoNP‐exposed, and CoCl_2_‐exposed skin) were preserved in RNAlater, and two biopsies (control and CoNP‐exposed skin) were fixed using 10% formalin and embedded in paraffin. To minimize the number of biopsies per participant, CoCl_2_‐exposed skin was not included for histological analysis. In total, five biopsies were collected from each participant.

All study subjects provided written informed consent. The study was conducted in accordance with the Declaration of Helsinki and approved by the ethical review board in Lund (no 2018/203), with biobank permission from Biobank Sweden (RBC South, no 2018‐A‐200, SC2802).

### 2.2 Histological Analysis of Biopsies

Formalin‐fixed paraffin‐embedded (FFPE) skin biopsies were cut into 4 μm thick sections and stained with hematoxylin and eosin (H&E) according to standard protocols. In brief, sections were stained with Harri's hematoxylin (PanReac AppliChem, Barcelona, Spain) for 6 min following deparaffinization and rehydration. After washing in running tap water, sections were briefly rinsed with 95% ethanol followed by staining with 1% alcohol‐based Eosin Y (Acros Organics, New Yersey, USA) for 1 min. Images of the tissues were acquired using Olympus IX73 microscope, image analysis was performed using ImageJ (version 1.52q) and figures were constructed in Adobe Illustrator (version 27.2).

Epidermal thickness was quantified in each biopsy by performing five independent measurements at distinct regions of the epidermis using ImageJ, and mean values were calculated for each sample. The association between epidermal thickness and clinical reaction strength was assessed using Pearson's correlation analysis.

### 2.3 Transcriptomics Data Generation and Analysis

RNA was extracted from skin biopsies stored in RNAlater using the AllPrep DNA/RNA/miRNA mini kit (Qiagen) according to the manufacturer's protocol. RNA purity and integrity were assessed using a Bioanalyzer (Agilent, Santa Clara, USA), and samples with RNA integrity number (RIN) values > 8 were considered suitable for further analysis. Library preparation and sequencing were performed by the National Genomics Infrastructure (NGI) in Stockholm, Sweden. In brief, libraries were prepared using the Illumina TruSeq Stranded mRNA (poly‐A selection) kit. Clustering was performed using the cBot system, and sequencing was carried out on one lane of the NovaSeq 6000 platform (NovaSeq Control Software 1.6.0/RTA v3.4.4) with a 2 × 151 bp setup using the NovaSeqXp workflow in S4 mode.

Quality control of raw sequencing reads was performed using FastQC (v0.11.8) [17], and adapter trimming and quality filtering were conducted using Trim Galore! (v0.5.0) [18]. Reads were aligned to the human reference genome using STAR (STAR_2.6.1a_08‐27) [19], and transcript assembly and quantification were performed using StringTie (v1.3.4d) [20].

Differential expression analysis was performed in the R environment (v4.3.2) using the DESeq2 package (v1.42.0) [21]. For comparison between CoCl_2_ and CoNP‐exposed samples, a paired design was implemented by including subject identity as a blocking factor in the model (design formula: ~subject + condition), thereby accounting for inter‐individual variability. Log2 fold change shrinkage was applied using the “apeglm” method [22]. Genes with an absolute fold change ≥ 1.5 and a Benjamini‐Hochberg adjusted *p*‐value < 0.5 were considered significantly differentially expressed. Data are available in the GEO database under accession number: GSE334401.

### 2.4 Gene Expression Data Visualization and Enrichment Analysis

Downstream analysis of gene expression data from skin biopsies was performed in the R environment. Unsupervized clustering using principal component analysis (PCA) implemented in the DESeq2 package (version 1.42.0) was used to visualize sample relationships. Associations between principal components and clinical reaction strength were assessed using Spearman's rank correlation.

Normalized expression values were obtained using variance stabilizing transformation (VST). Statistical comparisons of selected genes were performed using Student's *t*‐test or Wilcoxon rank‐sum test as appropriate, and *p*‐values < 0.05 were considered significant.

Gene set enrichment analysis (GSEA) was performed in R using the fgsea package, based on Hallmark gene sets obtained from the Molecular Signatures Database (MSigDB, version 7.5.1) [23]. Genes were ranked according to their correlation with reaction strength (Pearson correlation), and enrichment was assessed using a pre‐ranked approach.

Functional enrichment analysis of differentially expressed genes was performed using the clusterProfiler package (version 4.10.0) [24], including both Gene Ontology (GO) biological processes and Reactome pathway analysis. Data visualization was performed using ggplot2 (version 3.4.4) and ggpubr (version 0.6.0) [25, 26], and figures were assembled in Adobe Illustrator (v27.2). Venn diagrams were created using Venny 2.1 [27] and the VennDiagram [28] R package (version 1.7.3).

### 2.5 Deconvolution of Cell Type Abundance

Cellular compositional changes were explored through deconvolution of cell type abundance in the gene expression data from skin biopsies using the CIBERSORTx algorithm [29]. Estimation of leukocyte subset proportions was performed using the LM22 gene signature matrix, which is a validated signature of 22 individual immune cells [30]. For estimation of skin specific cell types, a custom cell signature was created using single‐cell gene expression profiles. To this end, 8 single‐cell RNAseq (scRNAseq) datasets from control skin sites were obtained (GSE147424) [31]. Clustering and assignment of cell types was performed as previously described [31] with few modifications. In brief, the Seurat package (version 5.0.1) [32] was used to integrate the scRNAseq data sets into a single Seurat object aligned by canonical correlation analysis for removal of batch effects. Cell type annotation was performed using the FindConservedMarkers function, and T cells were annotated using the SingleR [33] and the Human Primary Cell Atlas as the reference data set (http://biogps.org/dataset/BDS_00013/primary‐cell‐atlas/) [34], and concurrently evaluated manually. The annotated gene expression file was then used to create a custom signature matrix using CIBERSORTx. Estimations are based on 1000 permutations and a significance filter of *p* < 0.05 was applied to the estimated cell fractions.

### 2.6 Reconstructed Human Epidermal Model

An in vitro reconstructed human epidermal (RHE) model purchased from Episkin (Lyon, France) was used to assess the skin permeability of cobalt. To this end, models were exposed to 50 μL of CoNP or CoCl_2_ (nominal dose 1000 μg/mL) in sterile PBS on the air‐lifted side 17 days after seeding. Sterile PBS alone was used as a negative control. Exposures were performed for 12 h or 24 h. In brief, a rinse‐off sample was collected from the apical air‐facing side of the model by rinsing with 200 μL of sterile PBS three times. Tissue samples from the model and basal media were also collected for chemical analysis.

### 2.7 ICP‐MS Analysis

Cobalt content in samples collected from RHE model was analyzed by inductively coupled plasma mass spectrometry (ICP‐MS) using an Agilent 7800 ICP‐MS instrument (Agilent Technologies, Tokyo, Japan) equipped with an Agilent SPS 4 autosampler (Agilent Technologies, Tokyo, Japan). Samples were weighed into PFA tubes and digested using a mixture of 2 mL of 20% HNO_3_ (≥ 69%, Rotipuran Supra, Carl Roth, Karlsruhe, Germany) and 0.1 mL of H_2_O_2_ (30%, Suprapur, Merck, Darmstadt, Germany) with a 6‐h temperature program reaching temperatures up to 200°C. The resulting solutions were transferred to Falcon tubes and diluted with 8 mL of MiliQ H_2_O, resulting in approximately 5% HNO_3_ solutions ready for measurement. The isotopes ^59^Co and ^115^In (used as an internal standard) were monitored and recorded with an integration time of 0.1 s (10 replicates) in no‐gas mode. The instrument was equipped with nickel cones and operated at an RF power of 1550 W, using argon as both plasma gas (15 L/min) and carrier gas (1.05 L/min). Data evaluation was performed using the Agilent Mass Hunter software package (Workstation Software, Version C.01.06, 2019). Statistical significance was assessed by Wilcoxon rank‐sum test and *p*‐values of < 0.05 (Benjamini‐Hochberg adjusted) were considered significant.

### 2.8 NativeSkin Model

NativeSkin models (NSA11, Genoskin, Toulouse, France) generated from skin obtained from three donors were purchased and cultured using standard NativeSkin culture medium (Genoskin, Toulouse, France) at 37°C in a humidified incubator containing 5% CO_2_ according to the manufacturer's recommendations. Donor information on age, gender, skin type, allergies, and dermatological treatments was available. Following a 1‐h incubation, 20 μL of CoNP or CoCl_2_ (nominal dose 100 μg/cm^2^) in ultrapure water was topically applied to the skin surface. A negative control, exposed to 20 μL of ultrapure water only, was included. After 48 h of exposure, the skin biopsies were collected, excluding areas that had been covered by the silicone ring.

### 2.9 Immunostaining and LA‐ICP‐TOFMS Imaging of Skin Tissue Sections

Immunostaining and laser ablation inductively coupled plasma time‐of‐flight mass spectrometry (LA‐ICP‐TOFMS) measurements were performed as previously described [35]. In brief, thin 5 μm sections of formalin‐fixed paraffin‐embedded (FFPE) samples from the exposed NativeSkin models were stained using a cocktail of metal‐conjugated antibodies following deparaffinization, rehydration, antigen retrieval, and blocking. The antibodies used are listed in Table S1. Metal content and distribution measurements were then carried out using an Iridia 193 nm laser ablation (LA) system (Teledyne Photon Machines, Bozeman, MT, USA) coupled to an icpTOF 2R ICP‐TOFMS instrument (TOFWERK AG, Thun, Switzerland). The LA system comprised an ultrafast, low‐dispersion cell housed within a cobalt ablation chamber.

### 2.10 Single‐Cell Image Analysis

Image analysis at the single‐cell level of tissue sections from the NativeSkin models was performed using the MeXpose pipeline [35]. Two areas of equal size were selected from the tissue sections exposed to CoNP and CoCl_2_. The signal intensity maps generated by LA‐ICP‐TOFMS imaging were then processed using the pipeline. After cell segmentation and phenograph clustering, heatmaps were generated. Clusters were assigned to cell types based on their antibody expression, and tissue visualization of the clusters allowed confirmation of the correct cell type assignment. The intensities of intracellular cobalt and the expression of the DNA damage marker pH2Ax were then mapped for each cluster.

## 3 Results

### 3.1 Clinical Characterization of Patch Test Responses to CoNPs and CoCl_2_

The study included 14 individuals aged 43–74 years (median age 58.5) with a documented history of positive patch‐test reactions to cobalt. Further clinical characteristics are available in Table S2. Participants with active dermatitis or tattoos at the test site were excluded. Each subject was patch tested with CoNPs and CoCl_2_ at equivalent nominal cobalt doses (Figure 1A), as described by Midander et al. [12].

**FIGURE 1:**
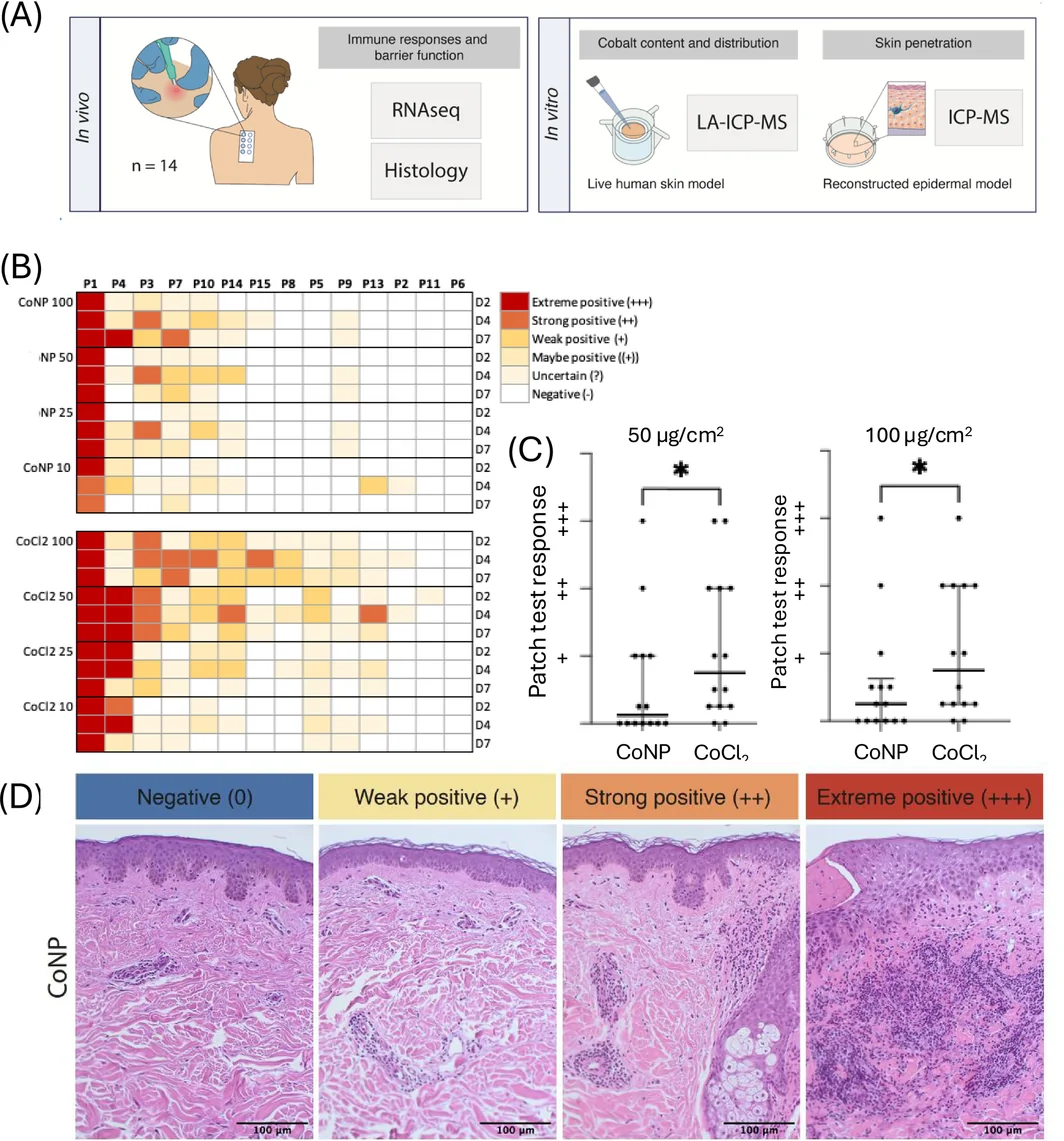
Study scheme, in vivo patch test results and skin tissue histology. (A) Overview of the analyzes performed in vivo and in vitro. (B) Positive patch test reaction readings (−, ?, (+), +, ++, +++) following CoNP or CoCl_2_ exposures (left panels), and statistical analysis of clinical reactions to CoCl_2_ compared to CoNP exposure (50 and 100 μg/cm^2^) on day 4. (D) Histological staining with H&E showing increased immune cell infiltration corresponding to the clinical evaluation in CoNP exposed skin. Data are presented as mean ± SEM; *n* = 14 patients; *p < 0.05, **p < 0.01 and ***p < 0.001, ****p < 0.0001. CoNP, cobalt nanoparticles; CoCl_2_, cobalt chloride; H&E, hematoxylin and eosin.

At the participant level, 10/14 individuals showed a positive reaction (≥ 1+) to CoCl_2_ and 7/14 to CoNPs. Seven participants reacted to both compounds, three reacted exclusively to CoCl_2_, and none reacted exclusively to CoNPs. One participant showed no reaction to either compound, while three exhibited only uncertain reactions without a clear positive response (Table S3).

Positive reactions to both substances were observed between days 2 and 7 (Figure 1B). Patch test responses were significantly stronger in CoCl_2_‐exposed skin compared with CoNP‐exposed sites at both day 2 and day 4 (Figures 1C and S1), whereas no significant difference was observed at day 7 (Figure S1). These findings indicate that CoNP‐induced skin reactions are generally milder during the early phase and exhibit slower development compared with CoCl_2_.

Histological analysis of cobalt‐exposed skin revealed pronounced perivascular immune cell infiltration (Figure 1D), along with increased epidermal thickness (Figure S2), the latter correlating with clinical reaction intensity.

To further evaluate the relationship between exposure dose and clinical outcome, we assessed the dose–response relationship on day 4 across the applied patch test concentrations (10–100 μg/cm^2^). For CoCl_2_, the proportion of positive reactions increased with dose, reaching 85.7% at 50–100 μg/cm^2^ compared to 64.3% at 10 μg/cm^2^. In contrast, CoNPs elicited a lower and less dose‐dependent response, with approximately 50%–57% of participants reacting across all tested concentrations. These findings indicate a steeper dose–response relationship for CoCl_2_, consistent with its greater skin penetration and bioavailability, whereas CoNP‐induced responses appear less sensitive to increasing nominal dose (Table S4).

### 3.2 Molecular Effects of CoNP and CoCl_2_ Skin Exposure

To investigate the molecular responses induced by cobalt nanoparticles (CoNP) and cobalt chloride (CoCl_2_) in the skin, RNA sequencing (RNA‐seq) was performed on biopsies collected from positive patch test reactions and control skin. Principal component analysis (PCA) of the transcriptomic data revealed substantial overlap in gene expression profiles between CoNP‐ and CoCl_2_‐treated samples (Figure S3). Notably, reaction strength emerged as the primary determinant of transcriptional variation, showing a strong association with the first principal component (PC1; Spearman's ρ = 0.90, *p* < 0.001) (Figure 2A). Samples with increasing clinical severity were distributed along PC1, while partial separation between CoCl_2_ and CoNP was observed along PC2 (14% variance explained).

**FIGURE 2:**
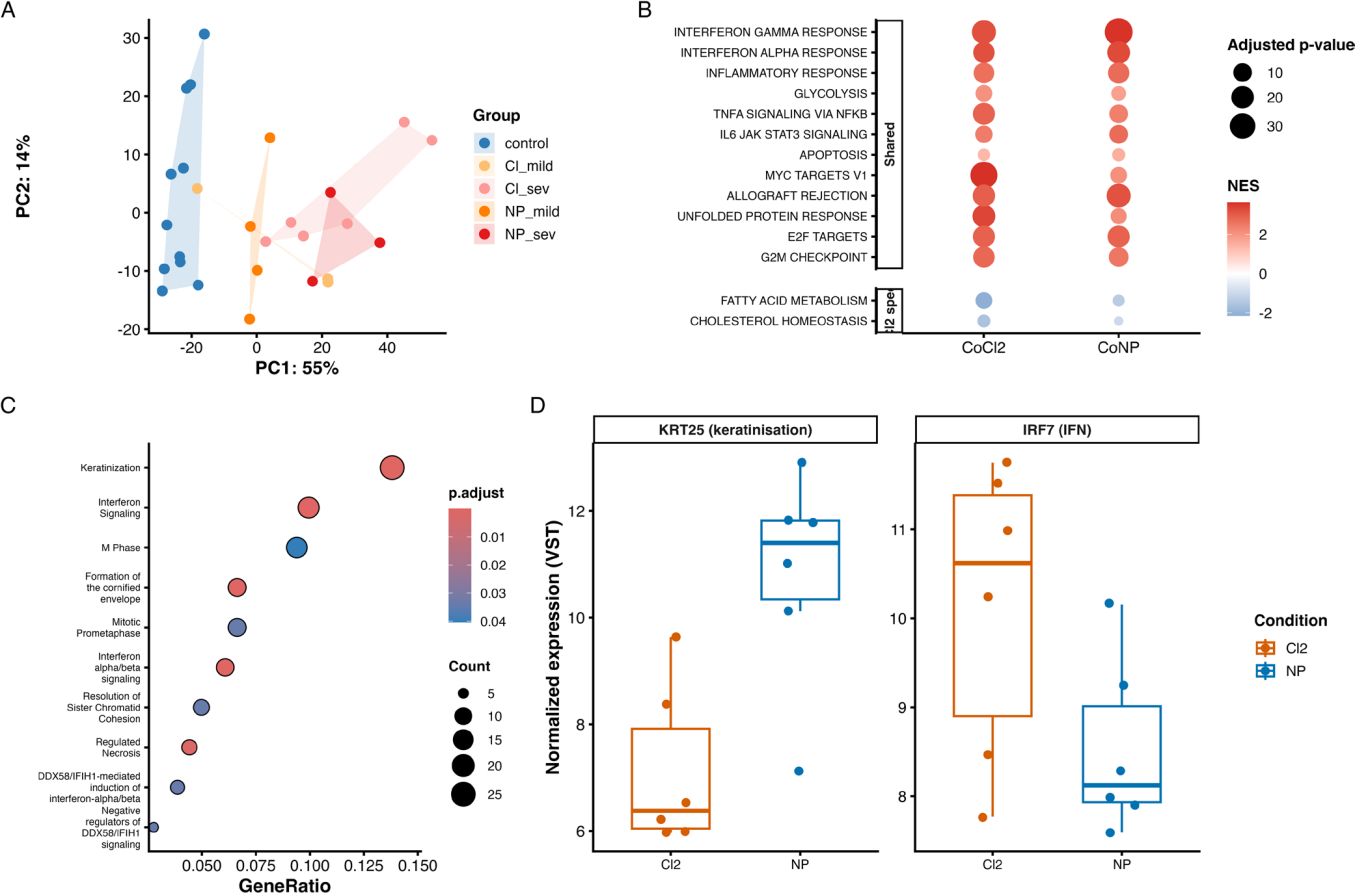
Reaction strength dominates transcriptomic variation, with distinct exposure‐specific signatures. (A) Principal component analysis (PCA) of variance‐stabilized RNA‐seq data from skin biopsies. Samples are colored by exposure and reaction strength. PC1 (55%) reflects increasing severity, while PC2 (14%) shows partial separation between CoCl_2_ and CoNP. (B) Hallmark gene set enrichment analysis (GSEA) based on correlation with reaction strength. Both exposures show enrichment of interferon signaling (IFN‐α/γ), inflammatory response, apoptosis, glycolysis, and cell cycle–related pathways. Lipid metabolism pathways are selectively enriched in CoCl_2_. Dot size indicates adjusted *p*‐value and color denotes normalized enrichment score (NES). (C) Reactome pathway enrichment of genes differentially expressed between CoCl_2_ and CoNP (paired DESeq2 analysis). CoCl_2_‐associated genes are enriched for interferon and immune signaling, whereas CoNP‐associated genes are enriched for keratinization and epidermal differentiation. Dot size reflects gene count and color indicates adjusted *p*‐value. (D) Representative genes illustrating exposure‐specific programs. IRF7 (interferon signaling) is higher in CoCl_2_, while KRT25 (keratinization) is higher in CoNP. Values represent variance‐stabilized counts.

Consistent with this, severe reactions were associated with a higher number of differentially expressed genes (DEGs) for both CoCl_2_ and CoNP exposures compared to mild reactions (Figure S4). Functional enrichment analysis of DEGs (Table S5) identified shared biological processes across both exposure types, including T cell activation, leukocyte cell–cell adhesion, and cytokine production (Figure S5, Tables S6–S9). Deconvolution analysis using CIBERSORTx further indicated increased estimated fractions of activated memory CD4^+^ T cells, proliferating keratinocytes, and vascular endothelial cells, alongside decreased resting mast cells, with increasing reaction strength (Figure S6). The estimated proportion of CD8+ T cells remained largely unchanged across conditions (Figure S6).

To assess pathway‐level changes associated with increasing reaction strength, gene set enrichment analysis (GSEA) using Hallmark gene sets was performed based on gene‐wise correlation with clinical severity. Both CoCl_2_ and CoNP exposures showed consistent enrichment of interferon signaling pathways (IFN‐α and IFN‐γ), inflammatory response, TNFα signaling via NF‐κβ, IL6‐JAK‐STAT3 signaling, apoptosis, and cell cycle‐related programs (Figure 2B, Tables S10 and S11). Glycolysis was also significantly associated with increasing reaction strength in both exposures, indicating a shared metabolic component of the inflammatory response.

In contrast, lipid metabolism pathways exhibited exposure‐dependent differences. Fatty acid metabolism and cholesterol homeostasis were significantly associated with reaction strength in CoCl_2_‐exposed skin, but not in CoNP‐exposed samples, indicating a more pronounced lipid metabolic reprogramming in response to CoCl_2_. These findings suggest that while the core inflammatory transcriptional program is largely shared, metabolic adaptations differ between the two cobalt forms.

To distinguish exposure‐specific effects, differential expression analysis comparing CoCl_2_ and CoNP was performed using a paired DESeq2 design including subject identity as a blocking factor. This analysis identified 281 significantly differentially expressed genes (adjusted *p* < 0.05) between exposures. Reactome pathway enrichment analysis of these genes demonstrated distinct transcriptional patterns: CoCl_2_‐associated genes were enriched for interferon signaling and immune‐related pathways, whereas CoNP‐associated genes were enriched for differentiation processes (Figure 2C). These exposure‐specific differences were further illustrated by representative genes (Figure 2D). Expression of *IRF7*, a key regulator of interferon signaling, was higher in CoCl_2_‐exposed samples, whereas *KRT25*, associated with keratinization, was higher in CoNP‐exposed samples.

Together, these findings indicate that reaction strength is the dominant driver of global transcriptomic variation in cobalt‐induced skin inflammation, characterized by a shared interferon‐driven inflammatory program across both exposures. In contrast, direct comparison between CoCl_2_ and CoNP reveals distinct exposure‐specific molecular signatures, with CoCl_2_ preferentially engaging interferon‐associated and antiviral pathways, and CoNP promoting keratinization and epithelial differentiation processes. Thus, while the overall inflammatory response is largely conserved, the two cobalt forms elicit divergent downstream programs, reflecting differential engagement of immune versus epithelial pathways.

### 3.3 Cobalt Chloride Exhibits Greater Skin Penetration Efficiency Compared to CoNPs

To evaluate the skin penetration capacity of CoNPs versus CoCl_2_, we used a reconstructed human epidermis (RHE) model under high‐dose topical exposure to assess cobalt retention and trans‐epidermal transport. Quantitative cobalt levels were determined by ICP‐MS in surface rinse‐off, tissue, and basal medium compartments (Figure 3A). Cobalt concentration is reported as molar values to allow direct comparison between liquid and tissue‐normalized measurements.

**FIGURE 3:**
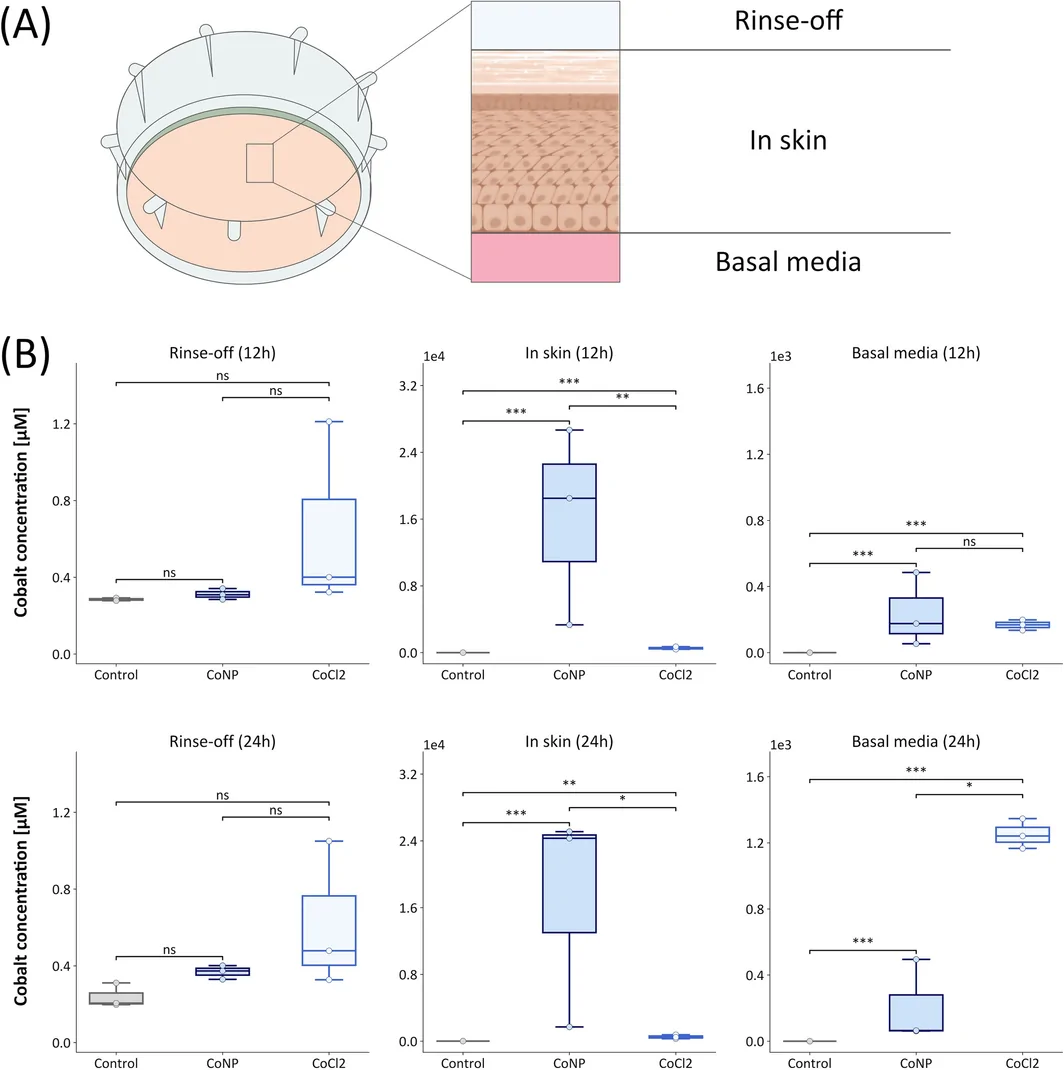
Compartmental distribution of cobalt in an RHE skin model. (A) Schematic of the RHE model compartments. (B) Cobalt concentrations in surface rinse‐off, epidermal tissue, and basal media at 12 h and 24 h. While CoNPs are largely retained in the skin, CoCl_2_ shows significantly higher flux into the basal media by 24 h. Boxplots show median and quartiles (*n* = 3). Statistical significance: *p < 0.05, **p < 0.01, ***p < 0.001; ns, non‐significant. CoNP, cobalt nanoparticles; CoCl_2_, cobalt chloride.

ICP‐MS results revealed distinct localization patterns of the two cobalt forms (Figure 3B). CoNP‐exposed tissue retained significantly higher total cobalt (Table S12), reaching ~17.0 mM at 24 h, compared to 0.5 mM for CoCl_2_. Given the applied dose and low tissue mass of the RHE model, local millimolar concentrations are expected. Notably, cobalt was nearly absent in the surface rinse‐off for both exposures at 12 and 24 h, indicating that the applied dose was almost entirely incorporated into the skin within the first hours of exposure.

Notably, CoNP levels stabilized early. Tissue concentrations (16.2 vs. 17.0 mM) and basal medium levels (0.2 μM vs. 0.2 μM) remained nearly identical between 12 and 24 h. This suggests that CoNPs are spatially confined within the tissue shortly after exposure, with no detectable redistribution over time.

In contrast, CoCl_2_ showed a much more dynamic penetration profile. While CoNP levels stabilized, CoCl_2_ concentrations in the basal medium surged between 12 and 24 h, increasing from 0.2 to 1.3 mM. These findings indicate that while CoCl_2_ requires an initial equilibration period to traverse the barrier, it eventually achieves significantly higher trans‐epidermal flux than CoNPs, which remain confined to the superficial layers.

### 3.4 Spatial Mapping Reveals CoNPs Primarily Localize to the Stratum Corneum

To determine whether the high tissue levels observed via ICP‐MS reflected actual uptake or merely surface trapping, we performed LA‐ICP‐TOFMS to spatially map cobalt in an ex vivo human skin model (NativeSkin) after 48 h of exposure, defining tissue architecture using structural biomarkers (Figure 4A).

**FIGURE 4:**
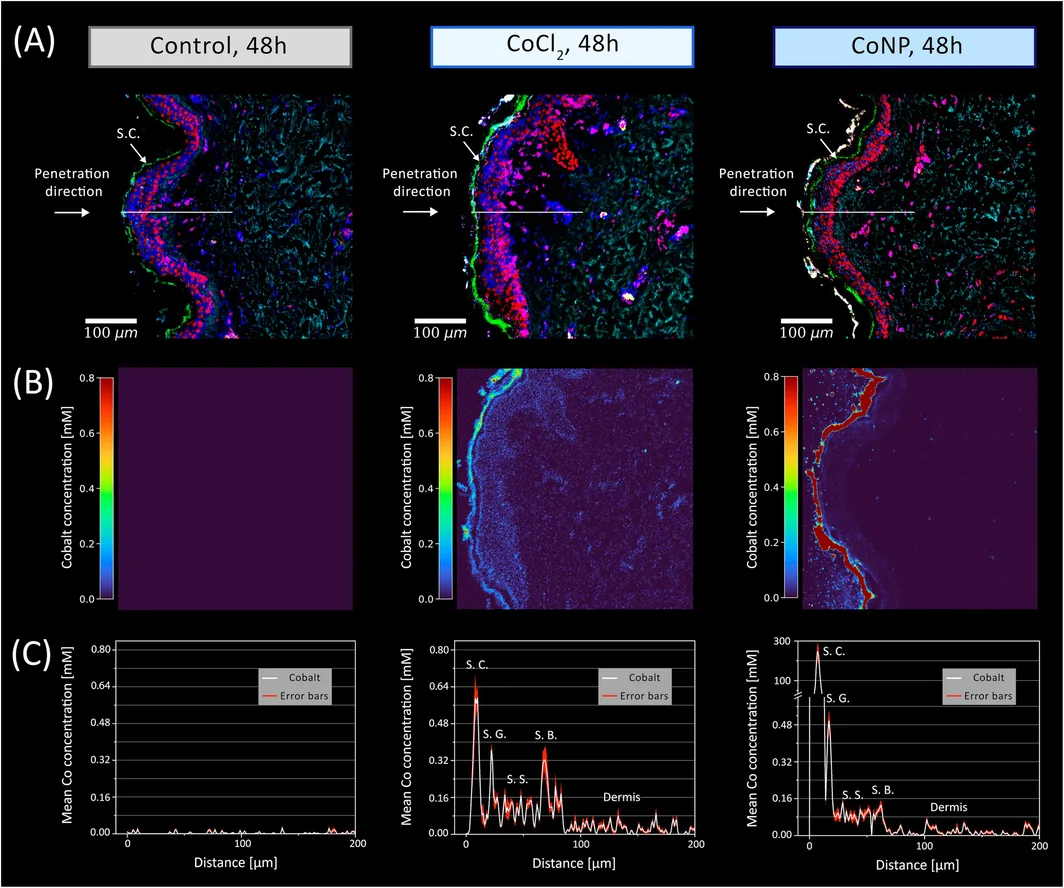
Spatial distribution and penetration depth of cobalt in ex vivo human skin (NativeSkin). (A) Tissue architecture of skin sections visualized through LA‐ICP‐TOFMS after 48 h exposure, with the following structural biomarkers: DNA Intercalator (red), CD44 (blue), Pan‐Keratin (green), α‐SMA (yellow), vimentin (magenta), and collagen type I (cyan). (B) Quantitative cobalt mapping illustrating that the high CoNP signal is driven almost exclusively by intense accumulation within the stratum corneum, whereas CoCl_2_ displays a deeper, more diffuse distribution profile. (C) Plotted line scan analysis showing mean cobalt concentration against distance. While CoNPs exhibit a peak signal in the stratum corneum nearly three orders of magnitude higher than CoCl_2_, they remain largely confined to the superficial layers. Conversely, CoCl_2_ demonstrates a significantly deeper penetration profile, with a distinct secondary peak in the basal layer (approx. 70 μm) and higher accumulation in the dermis. S.C., stratum corneum; S.G., stratum granulosum; S.S., stratum spinosum; S.B., stratum basale. CoNP, cobalt nanoparticles; CoCl_2_, cobalt chloride.

Consistent with the high skin retention observed in the RHE model, CoNP‐exposed skin displayed intense cobalt signals. Quantitative mapping revealed that this high content was driven almost exclusively by the stratum corneum (Figure 4B). Specifically, the stratum corneum contained a median concentration of 3.8 mM, decreasing sharply to 23.2 μM in the viable epidermis and 0.3 μM in the dermis (Figure S7). In contrast, CoCl_2_‐treated skin exhibited a lower total content but a deeper distribution profile, characterized by lower retention in the stratum corneum (0.8 mM) but significantly higher concentrations in the viable epidermis (72.7 μM) and dermis (8.5 μM).

Line scan analysis further confirmed a greater penetration depth for CoCl_2_ compared to the nanoparticles (Figure 4C). While the stratum corneum contained the peak signals for both exposures, CoNPs exhibited local maxima nearly three orders of magnitude higher than CoCl_2_. Although both profiles showed a similar peak in the granular layer (~20 μm), the deeper distribution diverged significantly: CoCl_2_ exhibited higher baseline levels in the spinous layer and a distinct secondary peak in the basal layer (~70 μm).

Figure 5 reveals distinct structural targets for each exposure. In CoCl_2_‐exposed skin, cobalt co‐localized with the vascular network, specifically accumulating within blood and lymphatic vessels. In contrast, this vascular association was absent for CoNPs; instead, nanoparticle accumulation was restricted to adnexal structures, such as the hair follicle and hair shaft, which may serve as pathways for localized deep penetration.

**FIGURE 5:**
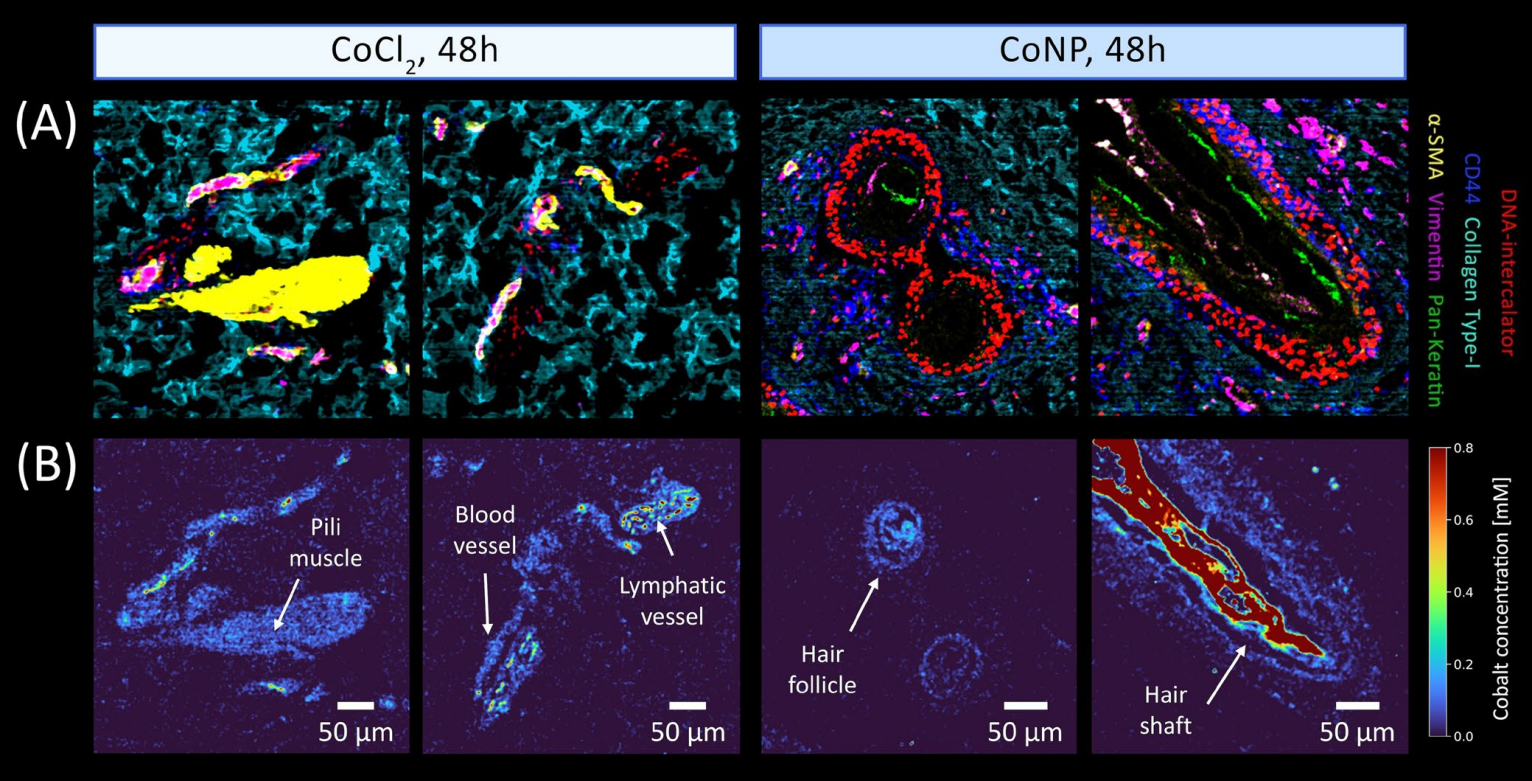
Localization of CoCl_2_ and CoNPs within specific skin structures. (A) Close‐up of ex vivo skin sections, tissue architecture visualized through LA‐ICP‐TOFMS using structural biomarkers. (B) Quantitative cobalt maps revealing distinct structural targets for each exposure. CoCl_2_ co‐localizes with the vascular network, accumulating in blood and lymphatic vessels, whereas CoNP accumulation is restricted to adnexal structures such as hair follicles and shafts. CoNP, cobalt nanoparticles; CoCl_2_, cobalt chloride.

A kinetic study of CoCl_2_ exposure (Figure 6) confirms a trend of progressive migration over 48 h. Initial exposure at 6 h shows cobalt confined primarily to the cornified layer. Between 24 and 48 h, the distribution shifts significantly deeper; as the cornified layer reaches saturation, cobalt accumulation intensifies within the viable epidermis and deeper dermal regions. By 48 h, high‐intensity hotspots appear in the basal region and the papillary dermis. This illustrates that by the 48 h mark, cobalt has permeated into the deeper anatomical structures of the skin.

**FIGURE 6:**
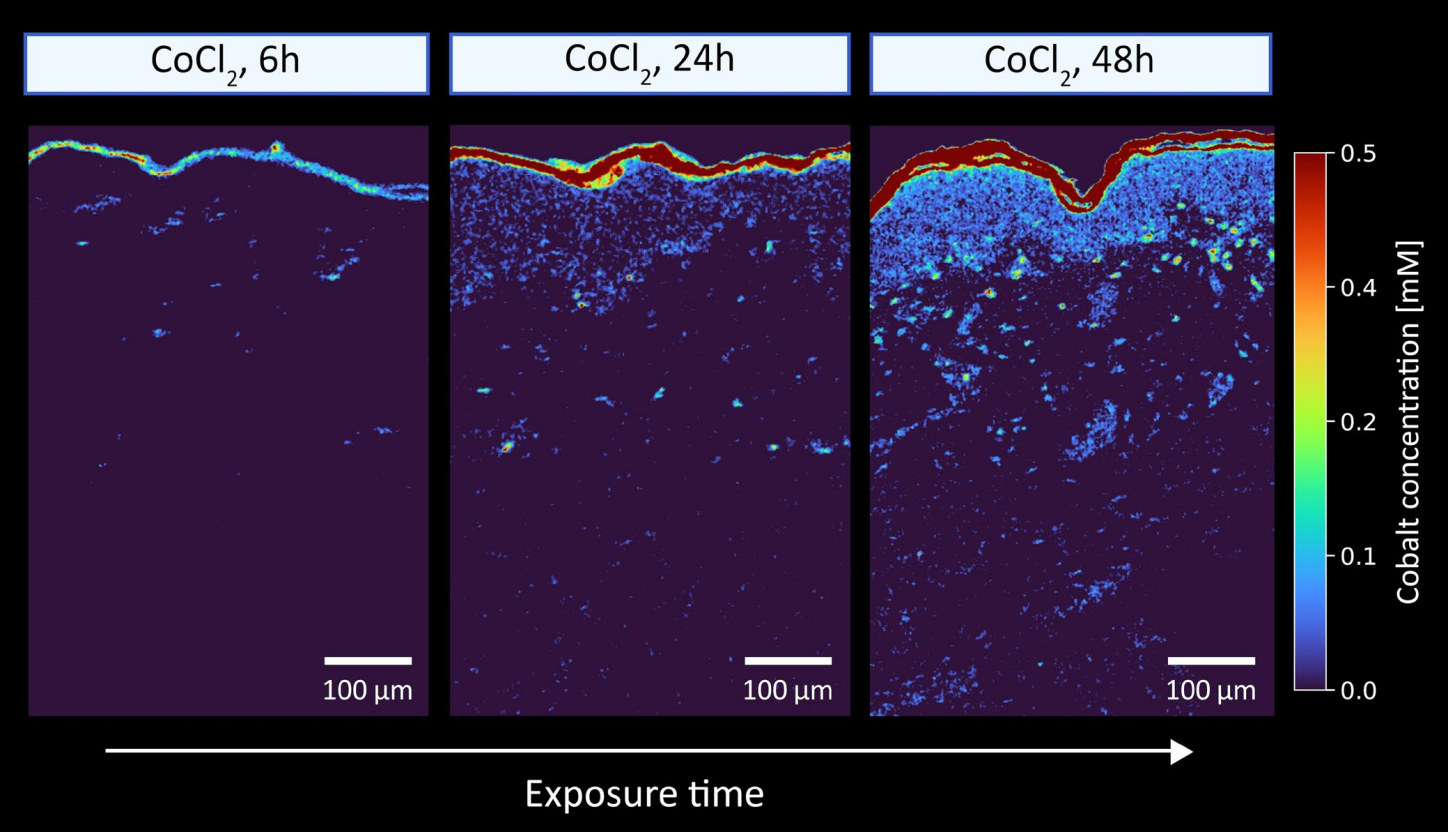
Time‐dependent penetration kinetics of CoCl_2_ in ex vivo human skin. Quantitative cobalt maps acquired by LA‐ICP‐TOFMS following CoCl_2_ exposure for 6 h, 24 h, and 48 h. At 6 h, cobalt is primarily confined to the cornified layer. Between 24 h and 48 h, cobalt migrates deeper as the cornified layer saturates, with accumulation intensifying in the viable epidermis and papillary dermis. By 48 h, high‐intensity hotspots in the basal region illustrate permeation into deep anatomical structures. CoCl_2_, cobalt chloride.

### 3.5 CoCl_2_ Exposure Leads to Greater Intracellular Cobalt Accumulation Compared to CoNPs

To assess intracellular cobalt distribution in skin tissue, we employed the MeXpose pipeline [35] on additional NativeSkin model sections. Single‐cell segmentation, utilizing CD44 as a surface marker and an iridium (Ir) nuclear intercalator, identified seven distinct cell populations: granular, spinous, and basal keratinocytes, fibroblasts, macrophages, vascular endothelial cells, and undefined dermal cells (Figure S6). This approach facilitated precise quantification and cell‐type specific mapping of cobalt uptake across different skin layers.

Following CoCl_2_ exposure, intracellular cobalt accumulation was evident in clusters expressing either pan‐keratin (epidermal marker) or collagen (dermal marker), indicating cobalt uptake across both compartments (Figure 7A). In contrast, cobalt uptake in CoNP‐exposed tissue was localized predominantly to keratinocyte clusters, with no appreciable accumulation detected in dermal cells, suggesting confinement to the epidermal layer.

**FIGURE 7:**
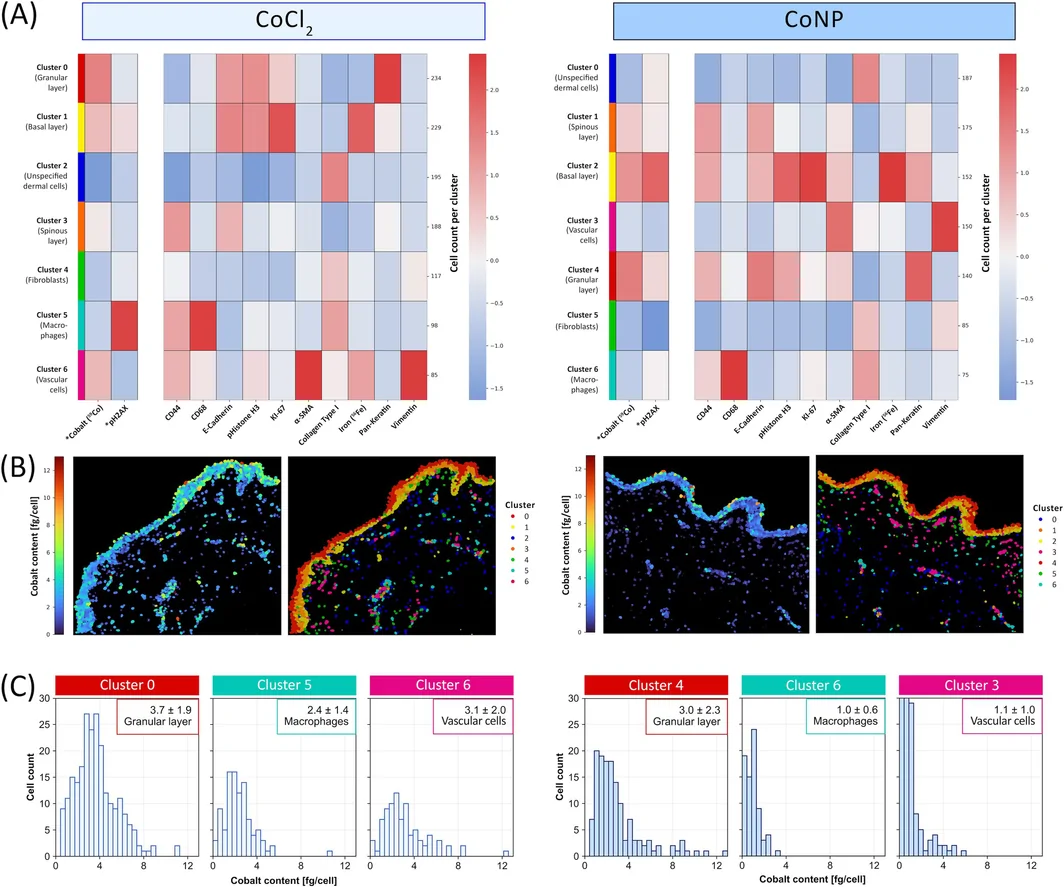
Analysis of cell‐specific cobalt uptake. (A) Heatmaps displaying expression patterns of assigned cell clusters alongside intracellular cobalt levels and the DNA damage marker γH2AX (pH2AX). (B) Visualization of intracellular cobalt (left) and tissue localization of identified phenotypic clusters (right) after single‐cell segmentation. (C) Quantitative histograms of cobalt content (fg/cell) in the granular layer, macrophages, and vascular cells. While superficial accumulation is comparable between treatments, CoCl_2_ leads to significantly higher cobalt levels in dermal populations. CoNP, cobalt nanoparticles; CoCl_2_, cobalt chloride.

Spatial distribution of intracellular cobalt was visualized by mapping cobalt‐rich cells to their corresponding phenotypic clusters (Figures 7A,B and S8). Corneocytes were excluded from this segmentation‐based analysis due to the absence of nuclear markers. Consequently, the substantial CoNP retention in the stratum corneum described earlier is not included here. Within the viable tissue, cobalt accumulation was visually pronounced in granular cells and actively proliferating epidermal cells (marked by high Ki‐67 and pHistone H3 expression) under both conditions.

However, quantitative analysis (Figure 7C) revealed a distinct depth‐dependent divergence in cell accumulation. A detailed view of the histograms for all clusters can be seen in Figure S8. While cobalt content in the superficial granular layer was comparable (3.7 fg/cell vs. 3.0 fg/cell), a clear divergence appeared in the spinous layer, where CoCl_2_ retention was markedly higher (2.8 vs. 1.8 fg/cell). In the proliferating basal layer, CoCl_2_ levels remained significantly elevated (2.8 vs. 2.2 fg/cell), though the differential was less pronounced than in the spinous compartment. In the dermis, CoCl_2_ exposure resulted in broad cobalt accumulation across multiple cell types. Fibroblasts contained 2.3 fg/cell compared to 0.7 fg/cell in the CoNP‐exposed samples, macrophages 2.4 versus 1.0 fg/cell, and vascular endothelial cells 3.1 versus 1.1 fg/cell. Statistical analysis confirmed that CoCl_2_‐treated samples exhibited significantly higher cobalt accumulation in these dermal populations (Table S13, Figures S9 and S10).

To assess cytotoxic responses across different cell populations, we analyzed expression of the DNA damage marker γH2AX (pH2AX). In CoCl_2_‐exposed tissue, while damage was evident in proliferating basal cells, expression was particularly pronounced in dermal macrophages (Figure 7A). Conversely, CoNP‐treated skin showed DNA damage confined primarily to the basal population, with minimal expression observed in differentiated keratinocytes or dermal macrophages. These findings indicate that cobalt‐induced cytotoxicity is compartment‐specific: CoCl_2_, likely due to high skin penetration efficiency, targets dermal immune cells in addition to the basal layer, whereas CoNP effects remain restricted to the proliferating basal epidermis.

## 4 Discussion

This study provides an integrated clinical and molecular characterization of cobalt nanoparticle (CoNP)–induced skin responses in cobalt‐sensitized individuals, directly compared with the conventional patch test allergen cobalt chloride (CoCl_2_). By combining in vivo patch testing with transcriptomic profiling and advanced human skin models, we delineate how the chemical form of cobalt influences skin penetration, immune activation, tissue targeting, and in vivo dose–response relationships.

High cobalt exposure may occur in occupational settings where cobalt‐containing materials are manufactured or handled. In addition to respiratory [36] and oral exposure [37], dermal contact with metallic surfaces represents a major route of exposure [3]. For example, hard metal discs containing 15% cobalt released 4.8 μg/cm^2^ cobalt when immersed in artificial sweat, whereas handling of the same discs under comparable conditions resulted in an average skin dose of 0.7 μg/cm^2^, indicating that only a fraction (approximately 15%) of the available cobalt is transferred to the skin during contact [38]. Importantly, elicitation thresholds in cobalt‐allergic individuals are considerably lower, with patch test studies reporting ED10 values between 0.0663 and 1.95 μg/cm^2^ [39]. These values overlap with skin doses measured in occupational settings, including the hard metal industry (median cobalt skin doses ranging from 0.011 to 1.51 μg/cm^2^) and among dental technicians (median range 0.0012 to 0.15 μg/cm^2^), suggesting that real‐world exposures may reach levels capable of eliciting contact dermatitis. In this context, the doses of cobalt chloride and nanoparticles applied in the present study were selected to span and exceed reported elicitation thresholds, thereby reflecting both typical and high‐end occupational exposure scenarios.

Cobalt is a well‐established and common contact allergen, with consistently high sensitization prevalence reported by surveillance networks such as the German Information Network of Departments of Dermatology (IVDK) [40]. While cobalt allergy has traditionally been linked to soluble ionic cobalt exposure, emerging evidence suggests that cobalt nanoparticles are also clinically relevant [41]. We and others have demonstrated that cobalt nanoparticles can elicit skin reactions in cobalt‐sensitized individuals under patch test conditions [12], supporting their capacity to trigger allergic contact dermatitis. From a real‐world perspective, exposure to cobalt nanoparticles may occur in several occupational settings where cobalt‐containing materials are handled or generated as fine particulate matter, including hard metal production, battery manufacturing and recycling, additive manufacturing, and dental or technical laboratory environments [42]. In these settings, dermal exposure to cobalt‐containing particles, including nanoscale fractions, may occur during powder handling, machining or recycling processes. The risk profile of CoNPs exposure may differ from ionic CoCl_2_ in that nanoparticles may act as a sustained source of bioavailable cobalt ions through gradual surface oxidation and dissolution, potentially prolonging cutaneous exposure. In addition, particulate cobalt may induce local inflammatory responses that enhance elicitation in sensitized individuals, whereas soluble CoCl_2_ provides immediate ionic exposure without a particular reservoir [12, 43]. These mechanistic differences may contribute to the partially distinct transcriptomic signatures observed in the present study despite broadly overlapping inflammatory responses.

The observed dose–response relationship supports the importance of cobalt speciation and bioavailability. While both CoCl_2_ and CoNP were applied at equivalent nominal doses, CoCl_2_ induced a clearer dose‐dependent increase in clinical reactivity, whereas CoNP responses remained comparatively stable across concentrations. This suggests that effective tissue dose, rather than applied dose, is a key determinant of biological response. The higher penetration efficiency of ionic cobalt likely results in greater intracellular exposure at increasing doses, thereby amplifying inflammatory signaling. In contrast, the retention of CoNPs within the stratum corneum may limit dose‐dependent increases in cellular exposure, leading to a blunted dose–response profile. These findings highlight that nominal exposure concentrations do not directly translate to biological effect, and underscore the importance of considering chemical form, penetration, and tissue distribution when assessing exposure risk.

Clinically, CoNPs elicited delayed and generally milder reactions compared with CoCl_2_, consistent with their more limited skin penetration. Despite these differences, both exposures induced highly overlapping transcriptional responses dominated by T cell activation, interferon signaling, and inflammatory pathways, indicating that the intensity of inflammation is a major determinant of the molecular profile. However, these responses must be interpreted in the context of differences in skin penetration and bioavailability between the two cobalt forms, which likely influence the magnitude and kinetics of the inflammatory response. These findings are in line with previous transcriptomic studies of contact allergens [44, 45], reinforcing the central role of the IFN–Th1 axis in allergic contact dermatitis and supporting the concept that reaction strength governs the magnitude of transcriptional change.

In addition to transcriptomic pathway analysis, cell type deconvolution using CIBERSORTx revealed dynamic changes in the cellular composition of inflamed skin. Increasing reaction strength was associated with higher estimated fractions of suprabasal and proliferating keratinocytes, vascular endothelial cells, and activated memory CD4^+^ T cells, together with a reduction in resting mast cells. These findings are consistent with a coordinated epithelial–immune response involving keratinocyte activation, vascular remodeling, and T cell–mediated inflammation [9, 46]. In contrast, CD8^+^ T cells were not selectively enriched in cobalt‐exposed skin, as supported by both deconvolution analysis and immunohistochemical evaluation, suggesting that cytotoxic T cell responses are not a dominant feature of this model.

At the pathway level, increasing reaction severity was associated with coordinated activation of interferon signaling (IFN‐α and IFN‐γ), TNFα/NF‐κβ signaling, IL6‐JAK‐STAT3 signaling, apoptosis, and cell cycle‐related programs. The enrichment of unfolded protein response and apoptotic pathways suggests that keratinocytes experience substantial cellular stress during cobalt‐induced inflammation, without providing direct evidence of oxidative stress as the primary driver. Activation of IL6‐STAT3 signaling together with enrichment of E2F targets and the G2M checkpoint is consistent with engagement of proliferative and regenerative programs [47], likely reflecting increased keratinocyte turnover in inflamed skin. Such coupling of inflammatory signaling and epithelial proliferation is a well‐recognized feature of barrier disruption and repair in inflammatory dermatoses.

In addition, glycolysis was significantly associated with reaction strength in both CoCl_2_ and CoNP‐exposed skin, indicating a shared metabolic reprogramming accompanying inflammation. Increased glycolysis is consistent with the metabolic demands of activated immune cells and proliferating keratinocytes, supporting cytokine production, biosynthesis, and tissue remodeling [48]. Together, these findings point to coordinated immunometabolic adaptation as part of the cutaneous inflammatory response.

In contrast to these shared severity‐driven programs, direct comparison of CoCl_2_ and CoNP exposures revealed distinct exposure‐specific transcriptional signatures. Differential expression analysis using a paired DESeq2 design identified 281 genes that differed significantly between exposures. Functional enrichment of these genes demonstrated that CoCl_2_ preferentially induced type I interferon and antiviral response pathways, exemplified by increased expression of IRF7, whereas CoNP exposure was associated with enrichment of keratinization and epidermal differentiation processes, including upregulation of KRT25. These findings indicate that, despite broadly similar inflammatory responses, the two cobalt forms engage distinct downstream programs, with CoCl_2_ driving a more immune‐dominant phenotype and CoNP promoting epithelial differentiation. These differences may, at least in part, reflect variation in effective tissue exposure, with ionic CoCl_2_ more readily penetrating the skin and inducing intracellular signaling, whereas CoNP may act through more localized or delayed mechanisms, including particle retention and gradual ion release.

CoCl_2_ exposure was also associated with altered lipid metabolic pathways, including fatty acid metabolism and cholesterol homeostasis, which were not significantly enriched in CoNP‐exposed samples. These findings suggest that ionic cobalt may exert a stronger impact on epidermal lipid composition and barrier‐associated metabolic processes, likely reflecting differences in skin penetration, intracellular bioavailability, and persistence, with ionic CoCl_2_ more readily accessing metabolically active epidermal layers compared to particulate CoNP. Given the central role of lipids in maintaining barrier integrity, such alterations may contribute to enhanced inflammation by facilitating allergen penetration [49] and amplifying immune activation [50, 51].

Importantly, the interpretation of exposure‐specific differences should be made with caution. The differential expression analysis comparing CoCl_2_ and CoNP did not explicitly account for variation in reaction strength, as this could not be incorporated into the paired model. Since CoNP‐induced reactions were generally milder, some of the observed differences may partly reflect residual differences in inflammatory intensity rather than purely exposure‐specific effects. Nevertheless, the consistent enrichment of interferon signaling in CoCl_2_ and keratinization in CoNP supports the presence of biologically meaningful divergence between the two exposure types. In addition, differences in penetration and persistence between cobalt forms may further contribute to these observed differences.

Previous studies on cobalt uptake have primarily relied on Franz diffusion cells or transmission electron microscopy to assess penetration and localization in the skin. Hagvall et al. [15] visualized the spatial distribution of cobalt ions within skin tissue. In our study, we employed ICP‐MS and LA‐ICP‐TOFMS to assess cobalt uptake and distribution using two human skin models. Using ICP‐MS, CoCl_2_ was detected not only within the epidermis but also in the dermis and basal culture medium, demonstrating efficient transepidermal permeation. Between 12 h and 24 h, cobalt levels in the basal medium increased substantially, whereas CoNP uptake remained largely confined to the tissue with little change over the same period.

LA‐ICP‐TOFMS mapping showed that CoCl_2_ gradually penetrated deeper into the viable epidermis and dermis over 6–48 h, with notable accumulation in the basal epidermis and papillary dermis by 48 h. CoCl_2_ was particularly enriched near vascular and lymphatic structures following CoCl_2_ exposure, implicating the cutaneous endothelium as an active participant in metal‐induced inflammation. Endothelial cells regulate leukocyte adhesion, transmigration, and cytokine production [52, 53], and their activation may facilitate the propagation or systemic dissemination of allergic responses. These findings add a new dimension to the pathophysiology of ACD by highlighting vascular–immune crosstalk as a potential modulator of reaction severity. The proinflammatory endothelial signatures observed here align with emerging transcriptomic evidence from psoriasis [52], suggesting that endothelial activation represents a shared hallmark of chronic inflammatory skin disease. Moreover, CoCl_2_ was enriched in dermal macrophages, where it correlated with cytotoxic effects. Dermal macrophages play a significant role in allergic contact dermatitis, acting as critical regulators of inflammation during both the sensitization and elicitation phases [54].

In contrast, CoNPs remained largely confined to the stratum corneum, with only minor cobalt detected in basal keratinocytes and no appreciable cobalt in the dermis, aligning with previous observations of metal nanoparticles [55]. This distribution pattern explains the delayed and less intense clinical reactivity to CoNPs but raises questions about follicular reservoirs as potential sites of prolonged or secondary exposure. Even relatively low levels of cobalt originating from retained or secondary sources, such as cobalt‐contaminated machine oil likely arising from metal wear during use, may elicit strong responses in sensitized individuals under certain exposure conditions [56], underscoring the need to account for nanoparticle retention and release kinetics in contact allergy risk assessment. The localization of CoNPs to adnexal structures also suggests possible routes for deeper dermal interaction over time, particularly under repeated or occluded exposure conditions.

Our results demonstrate that both ionic and nanoparticulate cobalt activate shared immune pathways but differ substantially in tissue targeting, penetration depth, and metabolic response. CoCl_2_ engages dermal immune and vascular networks, with higher intracellular accumulation in dermal macrophages and endothelial cells, while CoNPs primarily perturb epidermal keratinocytes and adnexal units. Recognizing these compartment‐specific immune responses may refine patch testing strategies, guide occupational safety evaluations for cobalt‐containing nanomaterials, and inform the development of targeted therapies aimed at IFN signaling or metabolic modulation.

The strengths of this study include in vivo patch testing combined with transcriptomic profiling and spatial metal imaging, providing an integrated framework to investigate how chemical form and tissue penetration shape allergic inflammation. However, several limitations should be considered. The cohort size is relatively small, which may limit statistical power and generalizability, and the evaluation of patch tests was not performed in a blinded manner. In addition, while the patch‐test exposure represents a controlled, continuous dermal surface loading, it may not fully reflect real‐world exposure dynamics such as intermittent contact, mechanical wear, or variable environmental conditions. Moreover, histological material was not available for CoCl_2_‐exposed skin, restricting direct comparison of tissue‐level cellular changes between exposures. Furthermore, while ICP‐MS provides precise quantification of total cobalt in skin compartments, it does not resolve spatial distribution or distinguish between ionic and particulate forms. Conversely, LA‐ICP‐TOFMS enables spatial mapping of cobalt within tissue sections but cannot differentiate cobalt ions from nanoparticles, nor provide information on associated chemical species or skin barrier integrity. These methodological constraints limit the ability to fully disentangle the relative contributions of cobalt form, localization, and bioavailability to the observed biological responses.

Future studies should aim to integrate higher‐resolution imaging approaches with histological and functional analyzes to better resolve cobalt speciation and tissue distribution. In addition, longitudinal studies are warranted to investigate nanoparticle persistence, vascular‐immune interactions, and the potential for targeting interferon or metabolic pathways in cobalt‐induced skin inflammation.

## 5 Conclusion

Cobalt‐induced ACD exemplifies how chemical speciation, tissue penetration, and immunometabolic context together shape allergic inflammation. Our data identify type I interferon signaling, cellular stress responses, endothelial activation, and metabolic adaptation as central mechanisms, while highlighting barrier disruption and differential tissue targeting as key modulators of disease expression. Beyond its relevance for cobalt allergy, this work establishes a framework for dissecting nanomaterial‐skin immune interactions, offering mechanistic insights that extend to broader contexts of environmental and occupational skin exposure.

## Author Contributions

Conception and design of the study: N.F., L.W., G.K. Acquisition of data: M.I., P.W., K.M., A.J., L.W., N.F., M.S., G.B. Analysis and interpretation of data: P.W., M.S., G.B., G.K., L.W., N.F. Drafting the article: P.W., M.S., N.F. Revising the article: M.S., K.M., A.J., M.I., N.F. Approval of the final version: all authors.

## Funding

This work was supported by Forskningsrådet om Hälsa, Arbetsliv och Välfärd (Grant 2018‐00601) and Työsuojelurahasto (Grant 113314).

## Conflicts of Interest

The authors declare no conflicts of interest.

## Supporting information


**Supporting information: S1.** supinfo/all70422‐sup‐0001‐Supinfo.pdf

## Data Availability

The data that support the findings of this study are openly available in Gene expression omnibus at https://www.ncbi.nlm.nih.gov/geo/.
