# Supplementary material for: Distinct Skin Penetration and Immune Responses to Ionic and Nanoparticulate Cobalt in Allergic Contact Dermatitis
Source: Allergy. 2026 Jun 20;81(10):3568–83. doi: 10.1111/all.70422 (PMC13646878; doi:10.1111/all.70422)
Supplement: Supplementary file 1 [file ALL-81-3568-s001.pdf]

Table S1. *List of metal-conjugated antibodies.*

Antibody target	Clone	Metal tag	Catalog number	Target
**α-SMA**	1A4	^{141}Pr	3141017D	Smooth muscle cells
**Vimentin**	D21H3	^{143}Nd	3143027D	Mesenchymal cells, fibroblasts
**Pan-Keratin**	C11	^{148}Nd	3148020D	Cornified layer/ keratinocytes
**CD44**	IM7	^{153}Eu	3153029D	Hyaluronic acid receptor /intercellular adhesion
**E-Cadherin**	24E10	^{158}Gd	3158029D	Epithelial cell membrane
**Collagen Type I**	Polyclonal	^{169}Tm	3169023D	Dermal collagen fibers
**CD68**	KP1	^{159}Tb	3159035D	Macrophages
**pH2AX**	S139	^{165}Ho	3165036D	DNA damage
**KI-67**	B56	^{168}Er	3168022D	Proliferation
**pHistone H3**	HTA28	^{176}Yb	3176024D	Mitosis

Table S2. *Clinical characteristics of cobalt-sensitized study participants.*

Patient ID	Age	Sex	Smoking	AD	Asthma	Rhinitis	Psoriasis	Other contact allergies	Sensitized to contact allergens
1	61	female	quit	yes	no	yes	no	yes	Au, Ni, Pd, Cr, Hg, Neomycin, Thiomersal, citraal, Amerchol L101
2	58	female	yes	yes	no	uncertain	yes	yes	Au, Cr, Neomycin, Ethylendiamine dihydrochloride, paratertiary butylphenolformaldehyde resin
3	51	female	quit	no	no	no	no	yes	Ni, Amerchol L101,
4	64	female	quit	no	no	no	no	yes	Au, Ni, Pd
5	70	male	no	yes	no	yes	uncertain	yes	Au, Ni, Cr, FMI
6	59	male	yes	no	no	no	no	yes	Ni, Au
7	57	female	no	no	no	yes	no	yes	Ni, parfym, preservatives
8	68	female	no	yes	yes	yes	no	yes	Ni, oakmoss
9	52	female	yes	yes	yes	yes	no	no	
10	61	male	no	no	no	no	no	yes	Cr, MI, sesquiterpene lactone mix, MCI/MI
11	67	male	no	no	no	no	no	yes	FMI, hydroxycitronellal, formaldehyde, quaternium 15, MCI/MI, neomycine, balsam of Peru, MCI
13	50	female	yes	yes	yes	yes	no	yes	Au, Ni, FMII, Lyral, Bisphenol F resin, Epoxi resin
14	43	female	no	yes	yes	yes	no	yes	Ni, Pd
15	51	female	no	no	no	no	no	yes	Ni, Pd

Table S3. *Summary of participant level responses.*

Response category	*n*
Positive* to $CoCl_2$ only (no CoNP response)	3
Positive* to CoNP only (no $CoCl_2$ response)	0
Positive* to both $CoCl_2$ and CoNP	7
No reaction to either compound	1
Uncertain reactions** only (no ≥1+ response)	3

*) Positive reactions defined as ≥1+ according to ESCD criteria.

**) "Uncertain only" indicates no reactions ≥1+ at any dose, but at least one doubtful or weak (uncertain) reaction

Table S4. *Dose response relationships.*

Exposure, day	Dose µg/cm2	Positive	TOTAL	%
CoNP D4	100	8	14	57
CoNP D4	50	7	14	50
CoNP D4	25	7	14	50
CoNP D4	10	8	14	57
$CoCl_2$ D4	100	12	14	86
$CoCl_2$ D4	50	12	14	86
$CoCl_2$ D4	25	10	14	71
$CoCl_2$ D4	10	9	14	64

Supplementary tables S4-S12 *are provided as Excel files.*

Table S5. Top differentially expressed genes in cobalt nanoparticle (CoNP) and cobalt chloride ($CoCl_2$)–exposed skin compared with reference skin.
Differential gene expression analysis was performed using RNA-seq data following exposure to CoNP or $CoCl_2$, with unexposed reference skin as comparator. Genes are ranked by adjusted p value. For each gene, the gene symbol (SYMBOL), Ensembl gene identifier (ENSEMBL), $\log_2$ fold change (log2FoldChange), standard error of the $\log_2$ fold change (lfcSE), Wald test statistic (stat), nominal p value (pvalue), and Benjamini–Hochberg adjusted p value (padj) are reported. Positive $\log_2$ fold change values indicate increased expression in exposed skin relative to reference skin, whereas negative values indicate decreased expression.

Table S6. Enrichment analysis of biological processes in the cobalt chloride ($CoCl_2$)-induced gene signature (Gene Ontology terms). Gene Ontology (GO) enrichment analysis was performed on genes associated with $CoCl_2$ exposure. Enriched biological processes are shown with corresponding enrichment statistics, including gene ratio, p value, and Benjamini–Hochberg adjusted p value (false discovery rate, FDR). Gene ratio represents the proportion of input genes assigned to each GO term. Terms are ranked by adjusted p value, and only significantly enriched terms ($FDR < 0.05$) are included.

Table S7. Enrichment analysis of biological processes in the cobalt nanoparticles (CoNP)-induced gene signature (Gene Ontology terms). Gene Ontology (GO) enrichment analysis was performed on genes associated with CoNP exposure. Enriched biological processes are shown with corresponding enrichment statistics, including gene ratio, p value, and Benjamini–Hochberg adjusted p value (false discovery rate, FDR). Gene ratio represents the proportion of input genes assigned to each GO term. Terms are ranked by adjusted p value, and only significantly enriched terms ($FDR < 0.05$) are included.

Table S8. Reactome pathway enrichment analysis of the cobalt chloride ($CoCl_2$)-induced gene signature. Pathway enrichment analysis was performed using the Reactome database on genes associated with cobalt chloride ($CoCl_2$) exposure. Enriched pathways are shown with corresponding enrichment statistics, including gene ratio, p value, and Benjamini–Hochberg adjusted p value (false discovery rate, FDR). Gene ratio represents the proportion of input genes assigned to each pathway. Pathways are ranked by adjusted p value, and only significantly enriched pathways ($FDR < 0.05$) are included.

Supplementary tables S4-S12 *are provided as Excel files, continued...*

Table S9. Reactome pathway enrichment analysis of the cobalt nanoparticle (CoNP)-induced gene signature. Pathway enrichment analysis was performed using the Reactome database on genes associated with CoNP exposure. Enriched pathways are shown with corresponding enrichment statistics, including gene ratio, p value, and Benjamini–Hochberg adjusted p value (false discovery rate, FDR). Gene ratio represents the proportion of input genes assigned to each pathway. Pathways are ranked by adjusted p value, and only significantly enriched pathways (FDR < 0.05) are included.

Table S10. Gene set enrichment analysis (GSEA) pathway summary and leading-edge genes for cobalt chloride ($CoCl_2$) and cobalt nanoparticle (CoNP) exposures. Gene set enrichment analysis (GSEA) was performed using Hallmark gene sets with genes ranked according to the Pearson correlation between variance-stabilized gene expression and clinical reaction severity (Reac) within each exposure. For each pathway, the normalized enrichment score (NES), nominal p value, and Benjamini–Hochberg adjusted p value (false discovery rate, FDR) are reported. Leading-edge genes represent the subset of genes contributing most strongly to the enrichment signal for each pathway. Pathways are ranked by adjusted p value, and positively enriched pathways (NES > 0) are shown.

Table S11. Genes associated with reaction severity in cobalt chloride ($CoCl_2$) and cobalt nanoparticle (CoNP) exposures. Genes were ranked within each exposure according to the Pearson correlation between variance-stabilized gene expression and clinical reaction severity (Reac). For each gene, the correlation coefficient (r), nominal p value, and Benjamini–Hochberg adjusted p value (false discovery rate, FDR) are provided. Positive correlations indicate increased gene expression with increasing reaction severity. Genes are ranked by correlation coefficient, and the top severity-associated genes for each exposure are shown.

Table S12. Comprehensive table containing individual replicate concentrations of the RHE experiments, group means (±SD), and comparative statistical analysis, including p-values (Excel-file “Supplementary_tables: S6”).

Table S13. Single-cell analysis raw data with cobalt content for each cell, mean values (±SD) for each phenotype, comparative statistical analysis, including p-values. (Excel-file “Supplementary_tables: S7”).

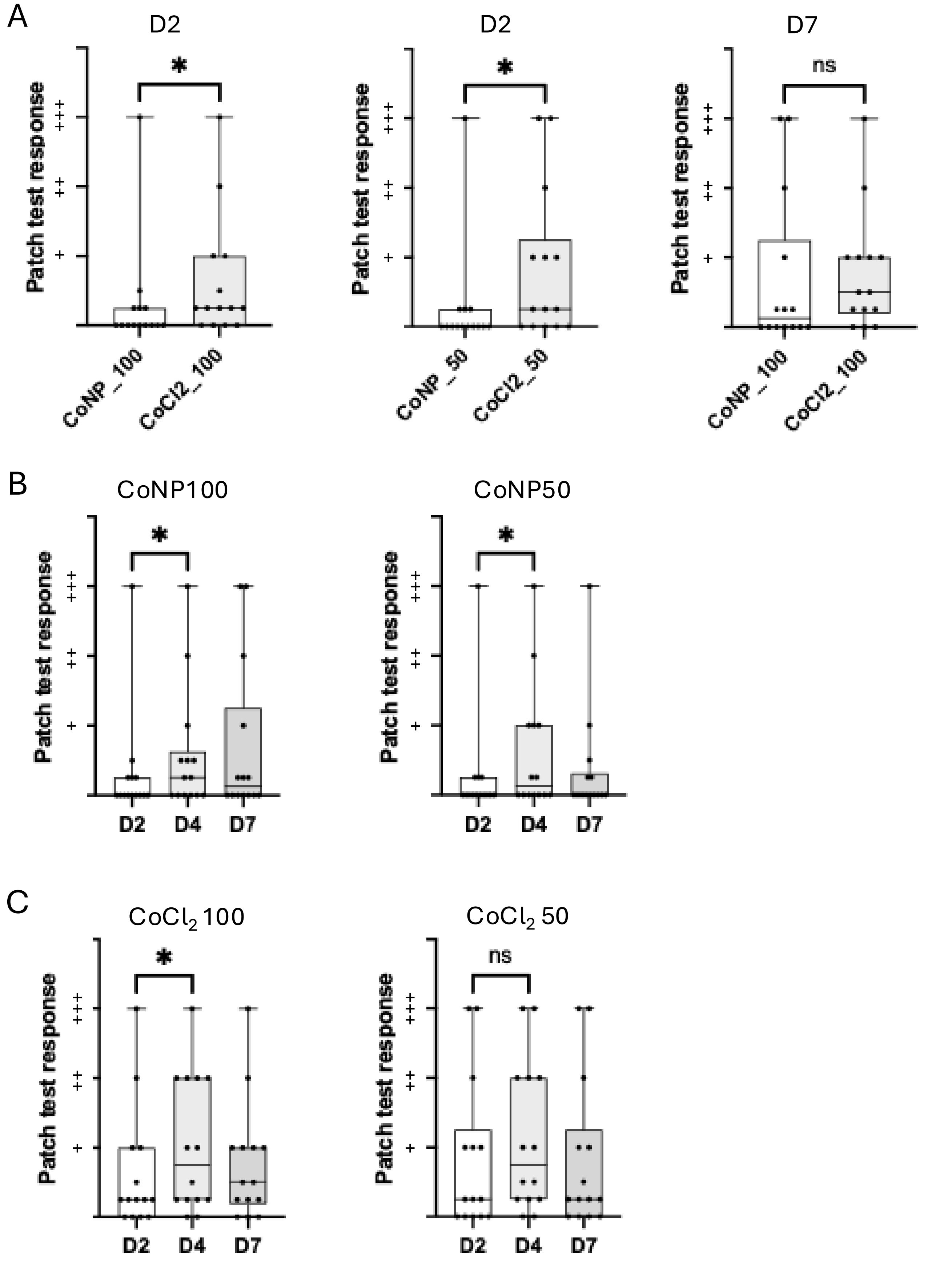

Figure S1. Patch test responses to CoNP and $CoCl_2$. (A) Patch test reactions were significantly stronger in $CoCl_2$-exposed skin compared to CoNP-exposed skin at day 2, but not at day 7. (B-C) CoNP responses increased over time, consistent with a delayed or slower development of reactivity compared with $CoCl_2$, although both substances elicited visible reactions within the same overall time window (days 2-7).

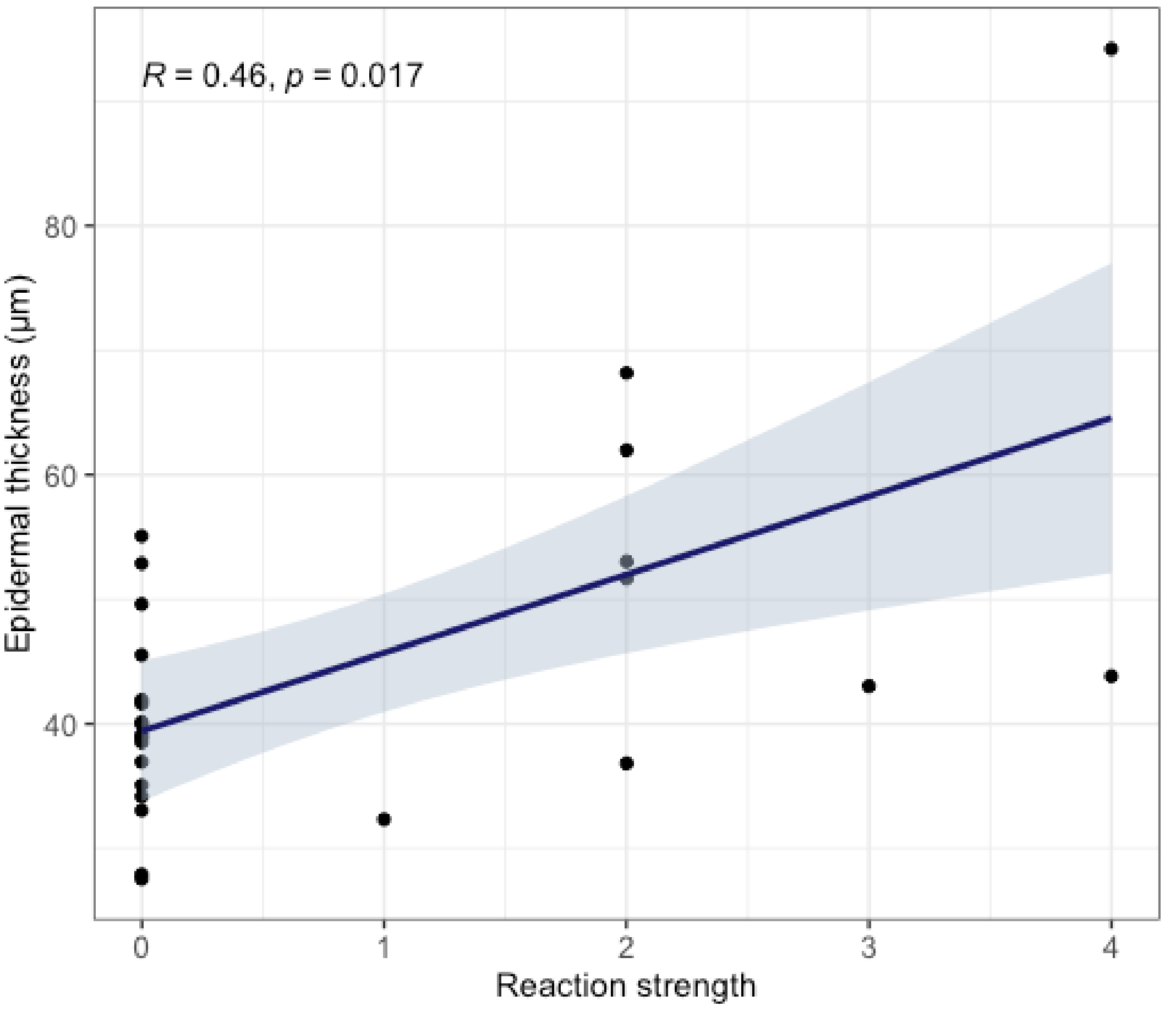

Figure S2. Association between epidermal thickness and patch test reaction severity following cobalt nanoparticle (CoNP) exposure. Epidermal thickness was quantified in skin biopsies obtained after CoNP exposure by performing five independent measurements across distinct regions of the epidermis for each sample, and mean values were used for analysis. Epidermal thickness (µm) is plotted against clinical reaction strength (ordinal scale). Each point represents an individual biopsy. The solid line indicates the linear regression fit, with shaded area representing the 95% confidence interval. A moderate positive correlation was observed between epidermal thickness and clinical reaction strength (Pearson's correlation coefficient $R = 0.46$, $p = 0.017$), indicating that increased clinical severity is associated with increased epidermal thickness. Pearson's correlation analysis was applied as both variables were treated as continuous or ordinal measures of increasing severity. No group-based statistical comparisons were performed, as the analysis was designed to assess continuous associations between histological and clinical parameters.

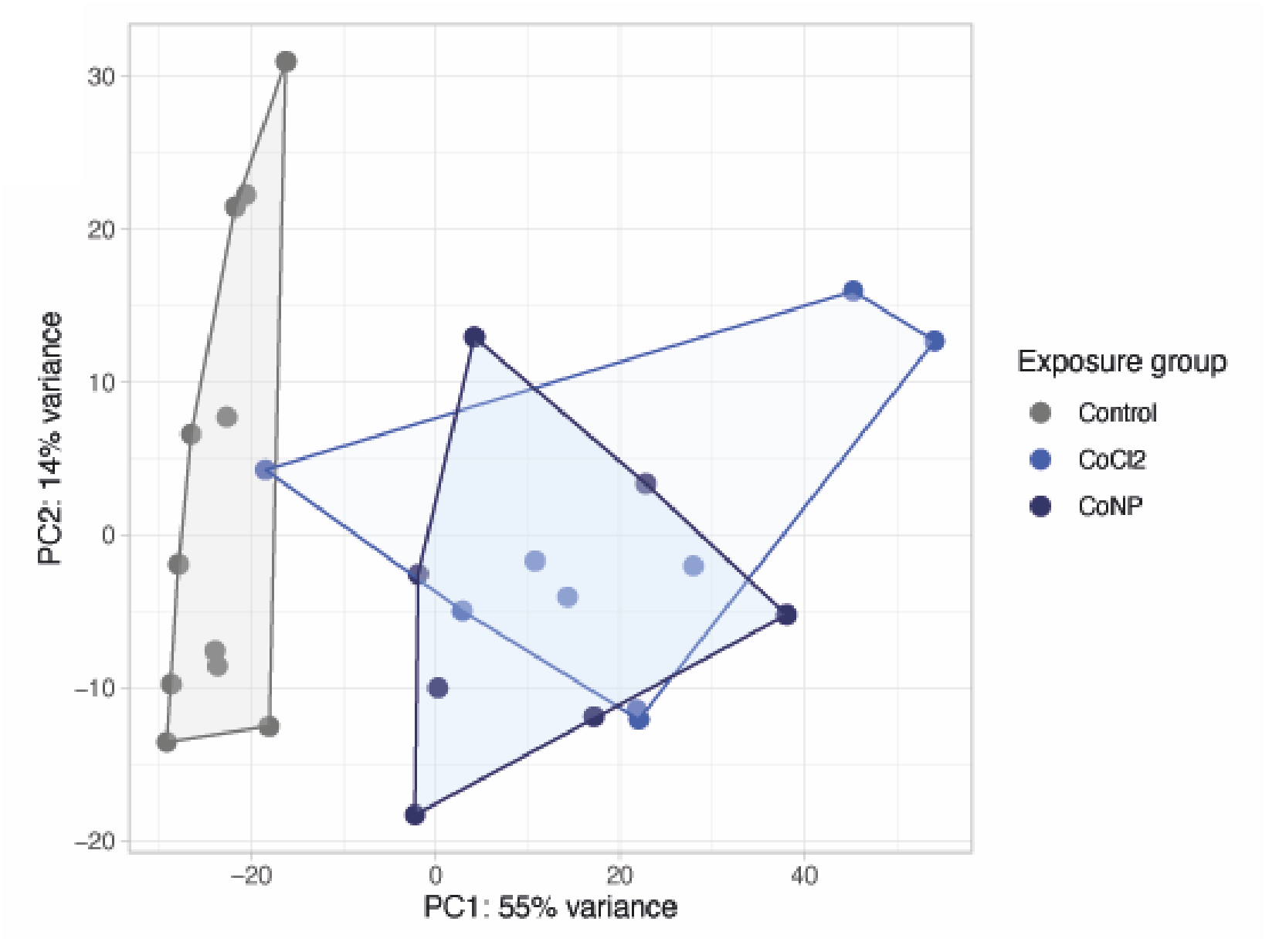

Figure S3. Principal component analysis (PCA) of transcriptomic responses following cobalt chloride ($CoCl_2$) and cobalt nanoparticle (CoNP) exposure. Principal component analysis was performed on variance-stabilized RNA-seq data derived from skin samples following exposure to cobalt chloride ($CoCl_2$) or cobalt nanoparticles (CoNP), with unexposed samples serving as controls. Each point represents an individual sample, colored by exposure group. The first two principal components are shown (PC1: 55% variance; PC2: 14% variance). Control samples cluster separately from exposed samples along PC1, indicating a global transcriptional shift following cobalt exposure. In contrast, $CoCl_2$- and CoNP-exposed samples show substantial overlap, with no clear separation between exposure types, suggesting broadly similar transcriptomic responses. Shaded polygons outline the distribution of samples within each group.

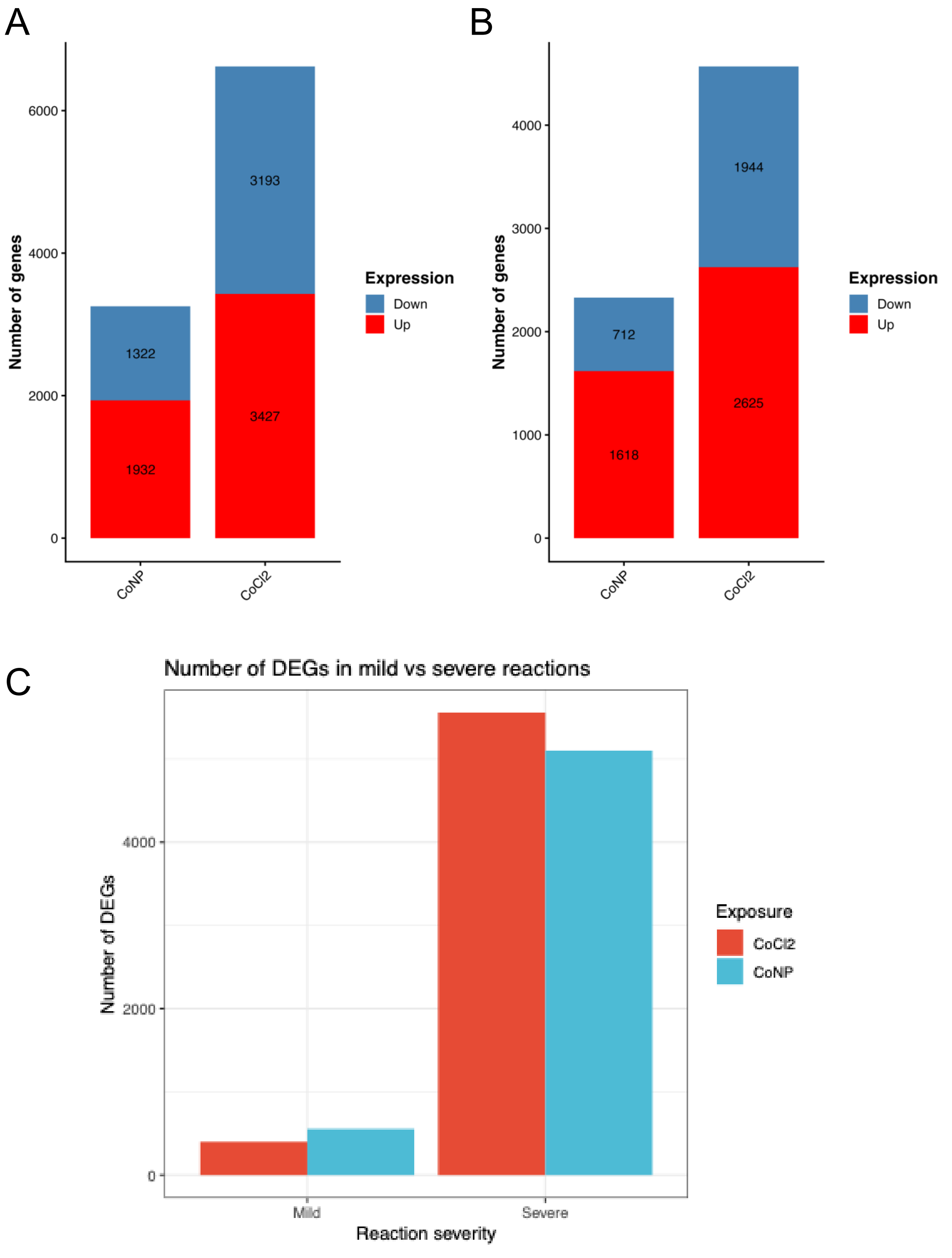

Figure S4. Differential gene expression following cobalt chloride ($CoCl_2$) and cobalt nanoparticle (CoNP) exposure. Stacked bar plots showing the number of upregulated and downregulated genes in skin exposed to $CoCl_2$ and CoNP compared with reference skin. (A) Differentially expressed genes (DEGs) identified using an adjusted p value < 0.05. (B) DEGs identified using an adjusted p value < 0.05 and an absolute $\log_2$ fold change ≥ 0.5. Upregulated genes are shown in red and downregulated genes in blue. (C) Number of DEGs associated with mild and severe clinical reactions for $CoCl_2$ and CoNP exposures. Bars indicate total DEG counts for each exposure and reaction severity category. Numbers within bars denote gene counts.

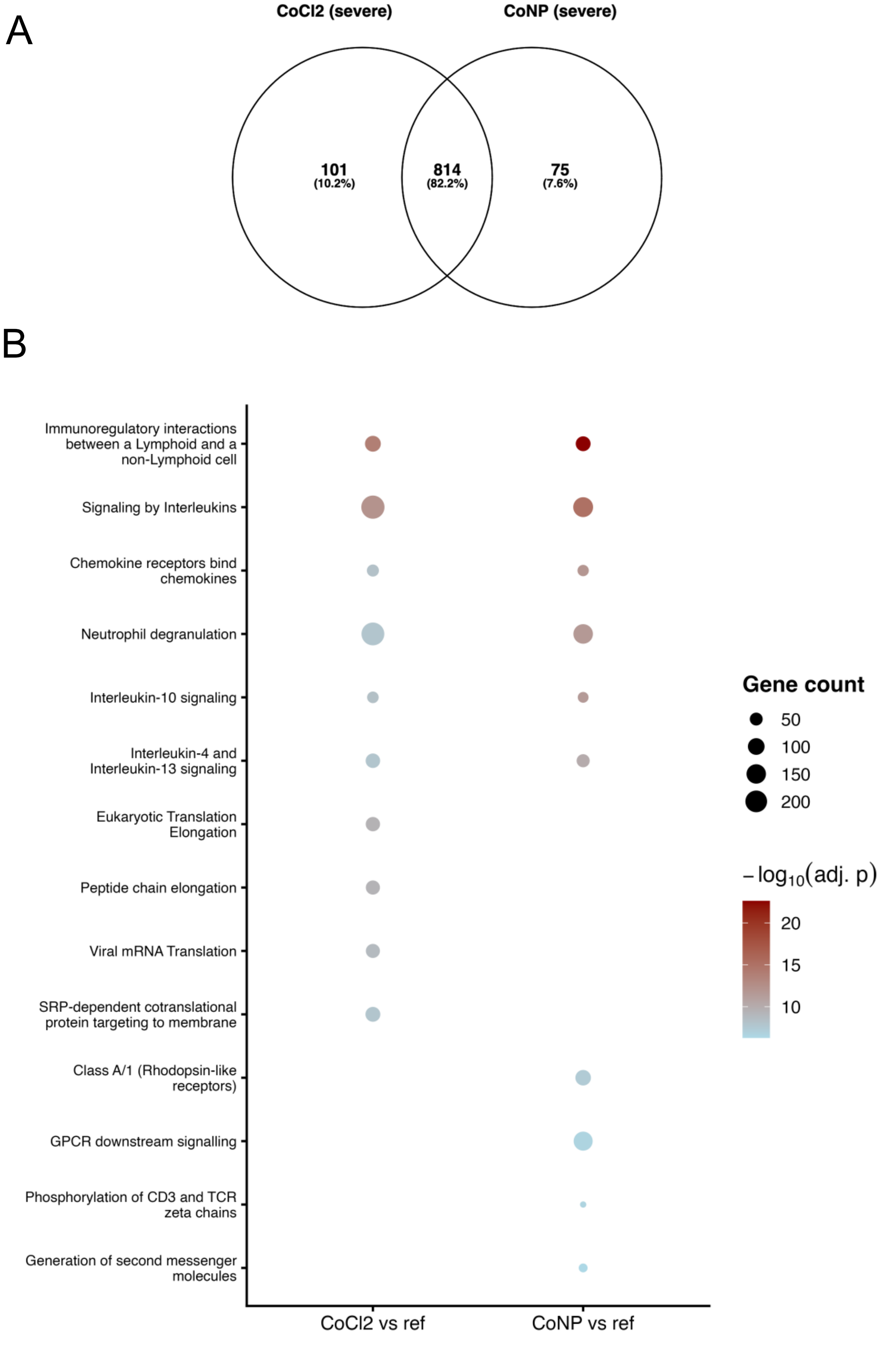

Figure S5. Overlap in enriched pathways between $CoCl_2$ and CoNP induced gene signatures. (A) Venn diagram of enrichments results (GO biological processes) of differentially expressed genes in severe clinical reactions to $CoCl_2$ and CoNPs. (B) Reactome pathway enrichment analysis of differentially expressed genes for $CoCl_2$ vs reference and CoNP vs reference. Both exposures show enrichment of shared immune and inflammatory pathways, indicating broadly similar transcriptional programs. Bubble size represents gene count and colour indicates –log10 adjusted p-value.

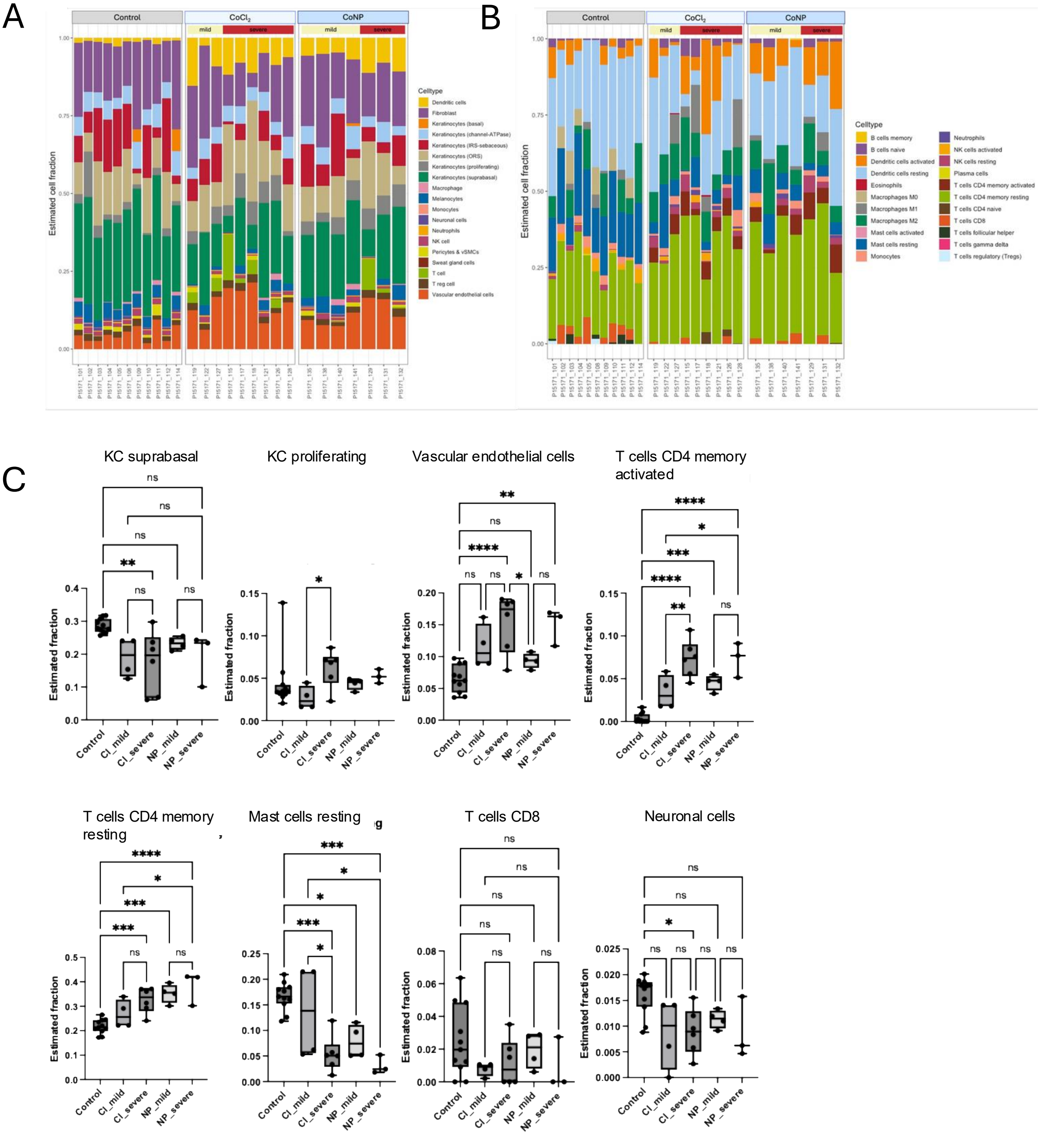

Figure S6. Leukocyte and structural cell deconvolution of cutaneous transcriptomes using CIBERSORTx. (A–B) Stacked bar plots showing the estimated relative fractions of cell populations inferred by CIBERSORTx from skin transcriptomic data across control and experimental groups ($CoCl_2$ and CoNP; mild and severe conditions). Panel A depicts structural and stromal cell types, while Panel B shows immune cell subsets. (C) Box-and-whisker plots summarizing the estimated proportions of selected cell populations across groups, including suprabasal keratinocytes, proliferating keratinocytes, vascular endothelial cells, $CD4^+$ memory T cells (activated and resting), resting mast cells, $CD8^+$ T cells, and neuronal cells. Statistical significance is indicated as follows: p<0.05, *p<0.01, **p<0.001, ***p<0.0001; ns, not significant.

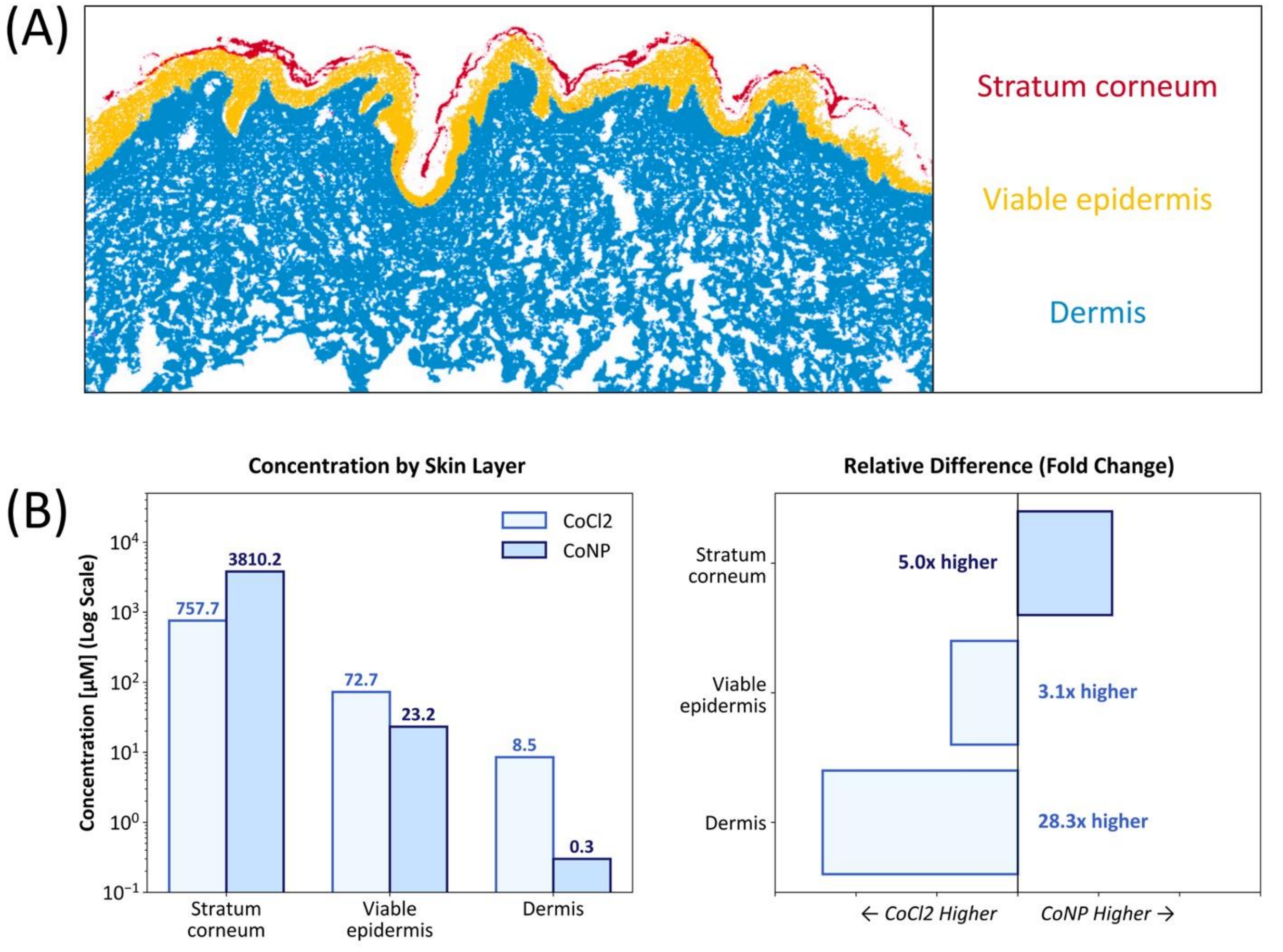

Figure S7. Compartmental distribution of $CoCl_2$ and CoNP in ex vivo skin model. (A) Representative segmentation of skin tissue measured by LA-ICP-TOFMS into the stratum corneum (red), viable epidermis (yellow), and dermis (blue). Segmentation was performed via K-means clustering (n=4) based on Pan-Keratin, Collagen Type I, and E-Cadherin markers. (B) Median cobalt concentrations (μM) and relative fold changes across identified layers for $CoCl_2$ and CoNP. CoNP, cobalt nanoparticles; $CoCl_2$, cobalt chloride.

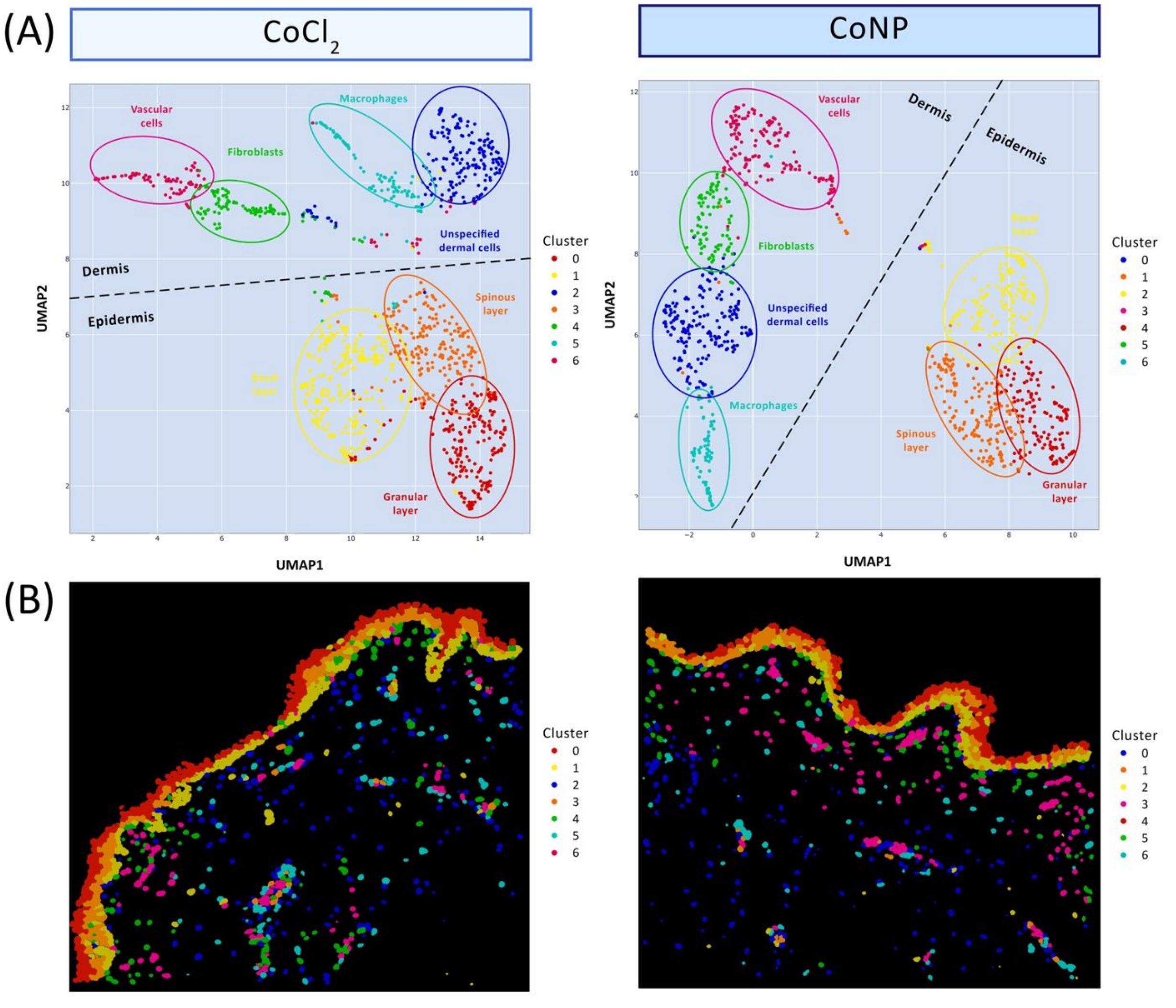

Figure S8. Characterization and spatial mapping of cellular phenotypes in human skin using the MeXpose pipeline. (A) UMAP plots of identified cellular clusters for $CoCl_2$- and CoNP-treated skin. Seven distinct clusters (0-6) were identified, revealing clear segregation between dermal cell populations and epidermal layers. (B) Spatial visualization of cellular clusters overlayed with the segmentation mask. This mapping confirms the accurate anatomical assignment of phenotypic clusters across the dermal-epidermal architecture. $CoCl_2$, cobalt chloride; CoNP, cobalt nanoparticles.

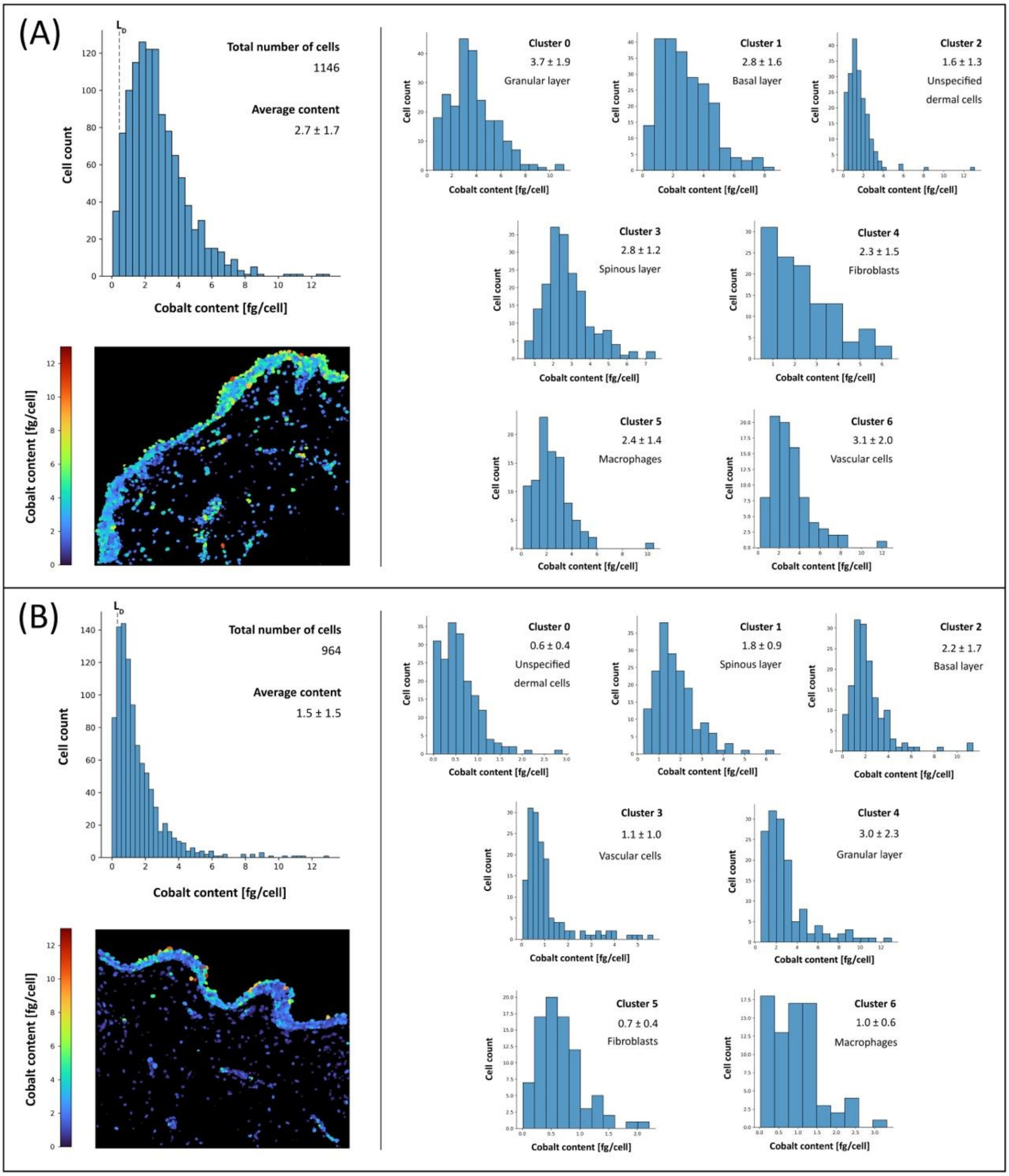

Figure S9. Quantitative single-cell analysis of cobalt accumulation across phenotypic clusters. Cellular cobalt content (fg/cell) was quantified for (A) $CoCl_2$ and (B) CoNP sections using the MeXpose pipeline. For each exposure, a global histogram representing the total cell population is displayed alongside a spatial distribution map and individual histograms for the seven identified clusters. CoCl2-treated cells showed significantly higher average cobalt accumulation (2.7 ± 1.7 fg) compared to CoNP (1.5 ± 1.5 fg), driven by 2-3 times higher uptake within dermal populations. $CoCl_2$, cobalt chloride; CoNP, cobalt nanoparticles; LD, limit of detection.

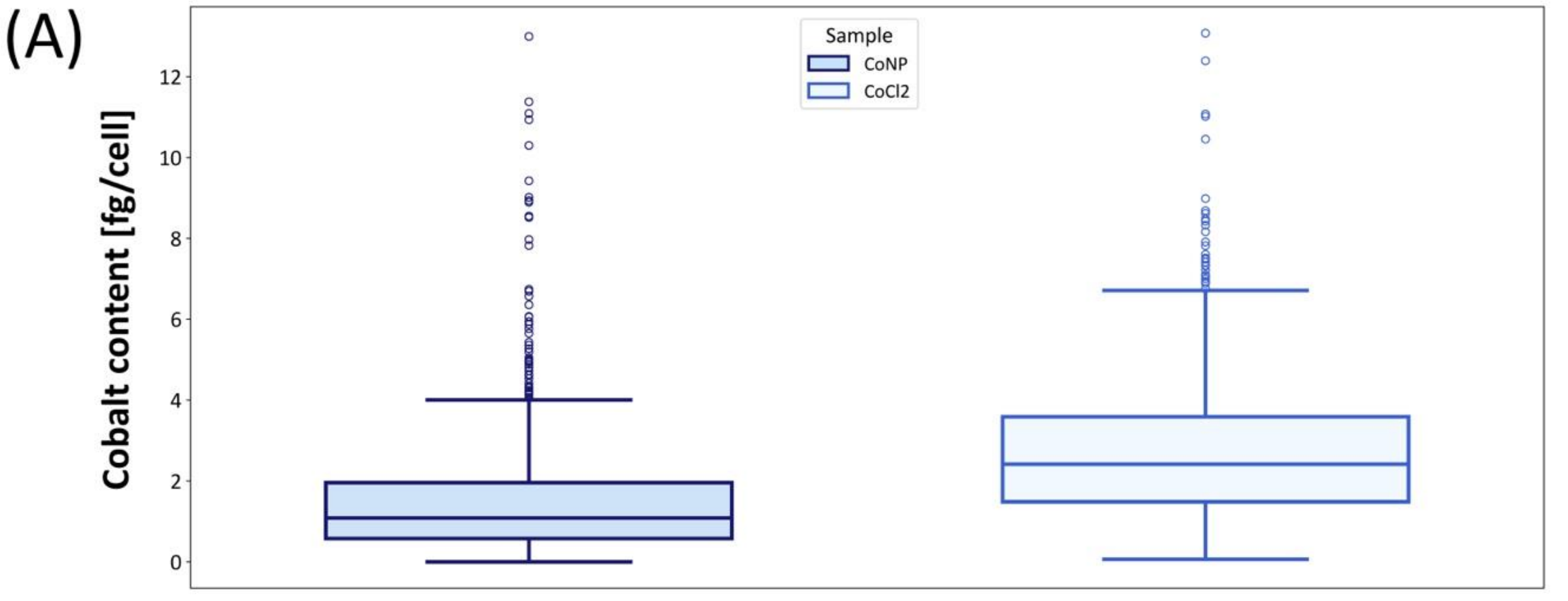

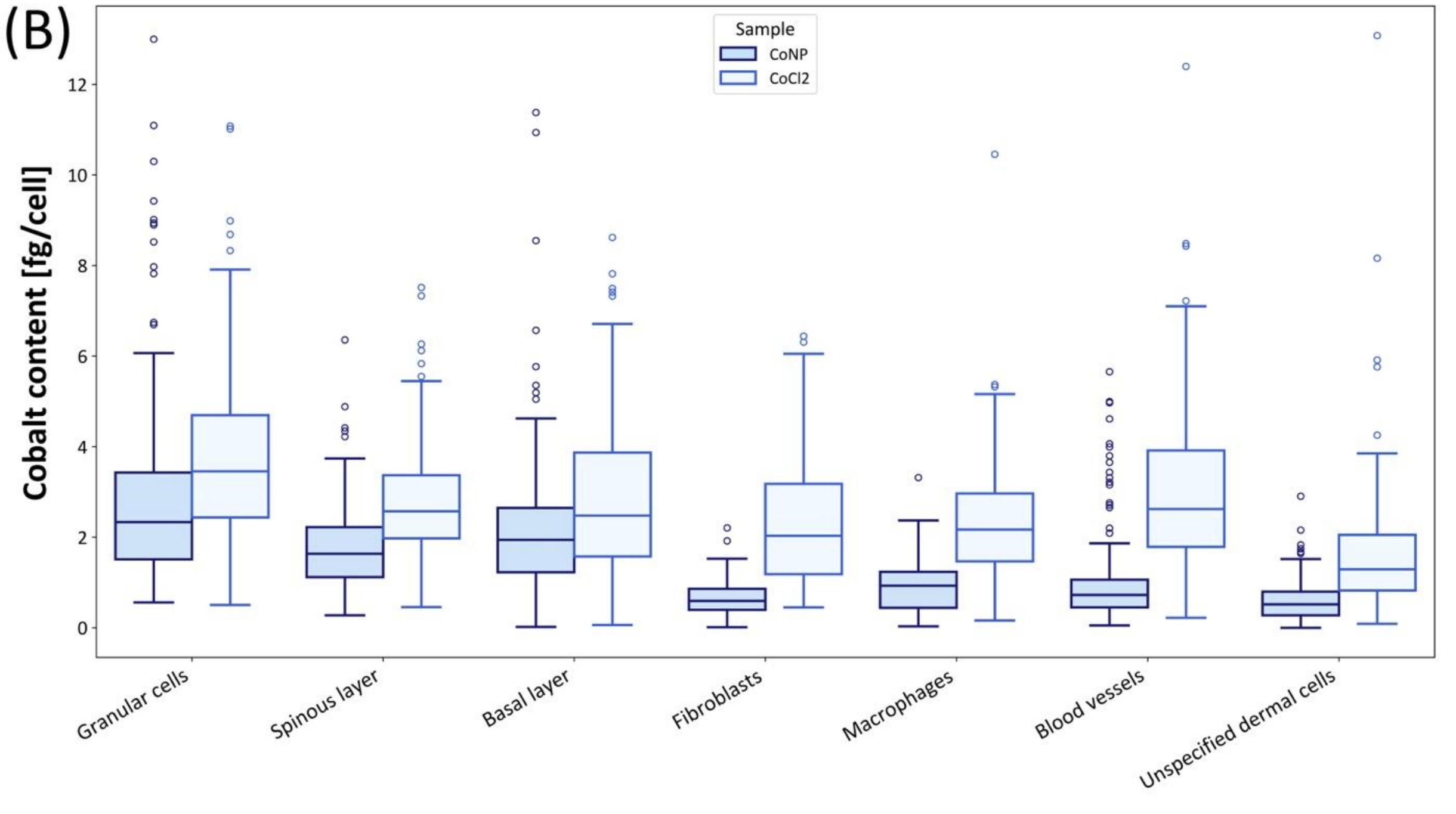

Figure S10. Comparative analysis of intracellular cobalt accumulation across cell populations. (A) Boxplots showing the cobalt content (fg/cell) for all segmented cells in $CoCl_2$ and CoNP samples. (B) Distribution of cobalt accumulation within individual cellular phenotypes. $CoCl_2$-treated cells demonstrate higher median intracellular cobalt levels compared to CoNP, with the most significant divergence occurring in dermal populations. Boxes represent the interquartile range (IQR) with median lines; whiskers indicate 1.5 x IQR, and individual outliers are plotted as open circles. $CoCl_2$, cobalt chloride; CoNP, cobalt nanoparticles.
